# Huntingtin Is Required for Neural But Not Cardiac/Pancreatic Progenitor Differentiation of Mouse Embryonic Stem Cells *In vitro*

**DOI:** 10.3389/fncel.2017.00033

**Published:** 2017-02-21

**Authors:** Man Shan Yu, Naoko Tanese

**Affiliations:** Department of Microbiology, New York University School of Medicine, New YorkNY, USA

**Keywords:** Huntington’s disease, huntingtin gene, embryonic stem cells, neural differentiation, embryoid bodies, neural stem cells

## Abstract

Mutation in the huntingtin (*HTT*) gene causes Huntington’s disease (HD). It is an autosomal dominant trinucleotide-repeat expansion disease in which CAG repeat sequence expands to >35. This results in the production of mutant HTT protein with an increased stretch of glutamines near the N-terminus. The wild type *HTT* gene encodes a 350 kD protein whose function remains elusive. Mutant HTT protein has been implicated in transcription, axonal transport, cytoskeletal structure/function, signal transduction, and autophagy. HD is characterized by the appearance of nuclear inclusions and degeneration of the striatum. Although HTT protein is expressed early in embryos, most patients develop symptoms in mid-life. It is also unclear why the ubiquitously expressed mutant HTT specifically causes striatal atrophy. Wild type Htt is essential for development as *Htt* knockout mice die at day E7.5. Increasing evidence suggests mutant Htt may alter neurogenesis and development of striatal neurons resulting in neuronal loss. Using a mouse embryonic stem cell model, we examined the role of Htt in neural differentiation. We found cells lacking Htt inefficient in generating neural stem cells. In contrast differentiation into progenitors of mesoderm and endoderm lineages was not affected. The data suggests Htt is essential for neural but not cardiac/pancreatic progenitor differentiation of embryonic stem cells *in vitro*.

## Introduction

Human HTT is a large protein of approximately 350 kDa made up of 3144 amino acids. It is nearly ubiquitously expressed with the highest level of expression in the brain and the testes. The size and predicted structure of the protein suggest its role as a protein scaffold that coordinates a multitude of cellular functions (reviewed in Saudou and Humbert, 2016). Studies have reported pro-survival properties of wild type Htt. It protects cells from cell death induced by a variety of stimuli including serum withdrawal (Rigamonti et al., 2000), mutant Htt (Leavitt et al., 2001), and NMDA receptor-mediated excitotoxicity (Leavitt et al., 2006). Htt has effects on gene transcription by binding to transcriptional regulators such as the repressor RE1-silencing transcription factor (Zuccato et al., 2003), p53 and CREB-binding protein (Steffan et al., 2000), NeuroD (Marcora et al., 2003), NF-κB (Takano and Gusella, 2002) and transcriptional activator Sp1 and co-activator TAF_II_130 (Dunah et al., 2002). Htt is involved in transport of a variety of vesicles including brain-derived neurotrophic factor-containing vesicles (Gauthier et al., 2004), synaptic precursor vesicles (Zala et al., 2013), and GABA receptor-containing vesicles (Twelvetrees et al., 2010). Htt coordinates cell division through the regulation of spindle pole assembly during mitosis (Godin et al., 2010; Elias et al., 2014). Moreover, Htt is reported involved in ciliogenesis (Keryer et al., 2011), endosomal trafficking (Pal et al., 2006), and autophagy (Kegel et al., 2000; Ravikumar et al., 2004).

Wild type Htt is essential for embryonic development as knockout of *Htt* in mice results in embryonic death at day 7.5 (Duyao et al., 1995; Nasir et al., 1995; Zeitlin et al., 1995). Htt may be needed for neurogenesis as reduced expression of wild type Htt causes impaired brain development and abnormal vascular morphogenesis in mice (White et al., 1997). Others reported cells without Htt can be differentiated into functional neurons (Metzler et al., 1999) or glial cells (Conforti et al., 2013). Thus, Htt’s role in neural development remains unclear.

Abnormal neurogenesis has been observed in HD. Increased cell proliferation and neurogenesis were found in human postmortem HD brains (Curtis et al., 2003), and in the quinolinic acid lesion rat model of HD (Tattersfield et al., 2004). Similarly, mutant Htt caused faster neuronal differentiation of embryonic and NSCs *in vitro* (Lorincz and Zawistowski, 2009). In contrast, reduced hippocampal neurogenesis was observed in R6/2 transgenic HD mice (Gil et al., 2005). Increasing evidence suggests mutant Htt causes dysregulated neurogenesis. In the HD R6/2 mice, expansion of striatal NSCs and altered migration of neural progenitor cells into the striatum were observed (Batista et al., 2006). A study reported that Q111 Htt knock-in mice (with glutamine repeats expanded to 111) exhibited defects in specification and maturation of striatal medium spiny neurons (Molero et al., 2009). Mutant Htt was also shown to affect cortical development by causing spindle misorientation in dividing cortical progenitors (Molina-Calavita et al., 2014). Selective expression of mutant Htt in mice up to postnatal day 21 resulted in impairment similar to mice expressing mutant Htt throughout life (Molero et al., 2016). Furthermore, mice expressing very low levels of Htt up to postnatal day 21 also exhibited late-life neurodegeneration phenotypes (Arteaga-Bracho et al., 2016). These studies suggest developmental abnormalities resulting from early mutant Htt expression or very low Htt expression may contribute to the pathogenesis of HD.

Neural stem cells derived from HD mice, or ES cells expressing mutant Htt or no Htt (*Htt-*null) have been studied for growth, motility, and biochemical properties (Ritch et al., 2012). Interestingly, NS cells derived from HD mouse brains showed reduced levels of cholesterol, increased reactive oxygen species, and impaired motility compared with NS cells from wild type animals, recapitulating phenotypes seen in HD patients. In contrast, *Htt* knockout (KO) NS cells derived from *Htt*-KO ES cells had increased cholesterol levels. Nguyen et al. used a neural induction culture model to examine stages of neural induction in ESCs expressing mutant Htt or in *Htt*-KO ESCs (Nguyen et al., 2013a). They reported disruption of proliferation, self-renewal, and the specification of NSCs in *Htt*-KO cells while mutant Htt enhanced neurogenesis. *Htt*-KO NSCs also showed increased endodermal and mesodermal gene expression suggesting Htt may be involved in neural and non-neural fate decisions. The same group also examined lineage differentiation of *Htt*-KO ESCs and ESCs expressing mutant Htt (Nguyen et al., 2013b). They found Htt required in the differentiation of ESCs into ectoderm, endoderm, and mesoderm while mutant Htt differentially impaired stage-specific developmental events.

Recent investigations on how mutant Htt affects neural development as well as the role of wild type Htt in neural development suggest neurodegeneration may result from both gain of toxic mutant Htt function and loss of wild type Htt function. In this study, we used the 5-stage differentiation protocol (Schroeder et al., 2009) to differentiate mouse ESCs lacking *Htt* to different cell lineages and examined the role of Htt in progenitor cell differentiation. We found Htt is required for ectoderm, but not mesoderm or endoderm differentiation under our experimental conditions.

## Materials and Methods

### Mouse Embryonic Stem Cell Culture

Four mESC lines used in this study are generous gifts of Dr. Scott O. Zeitlin (University of Virginia). They are: (1) R1, parental wild type ES cells; (2) *Htt*-null (HN), *Htt* nullizygous ES cells in which the promoter and exon 1 sequence of *Htt* were deleted (Zeitlin et al., 1995); (3) 7Q, 3xFlag-Htt^7Q/7Q^ ES cells that express wild type Htt Flag-tagged at the N-terminus; (4) 140Q, heterozygous 3xFlag-Htt^140Q/7Q^ ES cells carrying an *Htt* allele with an expanded polyQ tagged with a 3xFLAG tag at the N-terminus (Zheng et al., 2012).

Mouse embryonic stem cells were maintained undifferentiated on 0.1% gelatin-coated plates under feeder-free culture conditions in standard ES medium containing Dulbecco’s minimal essential medium (DMEM, Cellgro) supplemented with 15% ES-Cult FBS (STEMCELL Technologies), 1X Penicillin–Streptomycin–Glutamine (P/S/Q), 1 mM sodium pyruvate, 1X non-essential amino acids (NEAA), and 0.1 mM β-mercaptoethanol (all from GIBCO), 10^3^ Units/ml ESGRO mouse Leukemia Inhibitory Factor (LIF, Millipore), and 2 μM SU 5402 (VEGFR and FGFR inhibitor; Tocris Bioscience), 0.8 μM PD184352, and 3 μM CHIR99021 (MEK and GSK3 inhibitors, respectively, both from BioVision). Standard ES medium was changed daily and cells were passaged every 2–3 days using 0.05% Trypsin/EDTA.

### 5-Stage Neural Cell Differentiation

Mouse embryonic stem cells were differentiated into neural cells according to the 5-stage neural differentiation protocol developed by Dr. Ronald D.G. McKay (Okabe et al., 1996; Lee et al., 2000). Undifferentiated ES cells (Stage 1) were grown as described above for at least three passages before proceeding to the next stage.

To induce EBs formation (Stage 2), mESCs were dissociated into single-cell suspension with 0.05% trypsin/EDTA and plated onto 100 mm non-adherent bacterial petri dishes (2 × 10^6^ cells per dish) in the standard ES medium without LIF and the inhibitors. Floating EBs formed spontaneously were cultured for 4 days in suspension, collected and plated onto 100 mm tissue culture plates in the standard ES medium without LIF and the inhibitors.

After EBs attached to the culture plate began to differentiate (after 24 h), selection of nestin-positive cells (Stage 3) was initiated by replacing the standard ES medium with the serum-free ITSFn medium, which contained DMEM/Ham’s F-12 50/50 (Cellgro) supplemented with 1X P/S/Q, 5 μg/ml insulin, 50 μg/ml human apo-transferrin, 30 nM selenium chloride, and 5 μg/ml fibronectin. ITSFn medium was replenished every 2 days.

After 8 days of selection, expansion of nestin-positive cells (Stage 4) was initiated. Briefly, cells were dissociated with 0.05% trypsin/EDTA and plated on poly-L-ornithine and laminin-coated tissue culture plates or glass coverslips at a density of 1.5–2 × 10^5^ cells per cm^2^ in the N2 medium containing DMEM/Ham’s F-12 50/50 supplemented with 1X P/S/Q, 25 μg/ml insulin, 50 μg/ml human apo-transferrin, 30 nM selenium chloride, 20 nM progesterone, 100 μM putrescine, 10 ng/ml bFGF (R&D Systems), and 1 μg/ml laminin. Nestin-positive cells were expanded for 6 days. The medium was changed every 2 days.

Differentiation (Stage 5) was induced by withdrawing bFGF from the N2 medium. Cells were incubated under differentiation conditions for 6–15 days, changing the medium every 2 days.

### Alkaline Phosphatase Staining

Alkaline phosphatase staining was used to confirm undifferentiated mESCs. Cells cultured on 6-well plates were fixed in a fixative solution (citrate buffered acetone, 60%) for 30 s, and rinsed gently in deionized water. Cells were incubated in an alkaline-dye mixture, which contained Fast Blue RR Salt and Naphthol AS-MX Phosphate Alkaline Solution (Sigma-Aldrich) for 30 min at 37°C. Afterward, cells were rinsed thoroughly in deionized water, followed by examination under a microscope.

### Immunocytochemistry

Cells cultured on glass coverslips were fixed in 4% paraformaldehyde in PBS for 10 min, followed by permeabilization in PBS containing 0.25% Triton X-100 for 10 min. Blocking was carried out by incubation in 1% BSA in PBST (PBS + 0.1% Tween 20) for 30 min. Cells were incubated with primary antibodies for 2 h at room temperature. Following antibodies were used at indicated dilutions: mouse α-nestin (Millipore; 1:300), mouse α-β-III tubulin (Millipore; 1:400) and rabbit α-GFAP (Sigma-Aldrich; 1:400). Fluorescently labeled goat α-mouse/rabbit secondary antibody (1:500 for 1 h at room temperature) was applied to samples in the dark. Nuclear counterstaining was performed by incubating samples in 1 mM TO-PRO-3 (Invitrogen) in PBS for 20 min followed by confocal imaging.

### Three Germ Layer Differentiation via Hanging-Drop EB Formation

Htt mESC lines were induced to differentiate into three germ layers (ectoderm, mesoderm, and endoderm). Differentiation protocols used involved hanging-drop EB formation (Schroeder et al., 2009) in which a defined number of mESCs was used to form one EB. In this way, all EBs generated would be of similar size. To make hanging-drop EBs, mESCs were maintained undifferentiated for at least three passages, followed by trypsinization and counting. Single-cell suspension containing defined mESC number (200, 500, and 600 cells/20 μl for neural, cardiac, and pancreatic differentiation, respectively) was prepared in the Differentiation Medium I consisting of Iscove’s modification of DMEM (IMDM) supplemented with 20% ES-Cult FBS, 1X P/S/Q, 1 mM sodium pyruvate, 1X NEAA and 450 μM monothioglycerol (Sigma-Aldrich). On the lid of a 100 mm bacteriological Petri dish, about 60 droplets (20 μl per drop) of mESC suspension were placed. 10 ml of PBS was added to the dish. The lid was inverted and mESCs in hanging-drops were cultivated for 2–3 days. During this period, mESCs aggregated to form one EB within each droplet. Hanging-drop EBs were then incubated in suspension in the Differentiation Medium I for 2–3 days until the time of specific lineage differentiation. When collecting or transferring floating EBs, large orifice 200 μl tips were used to keep EBs intact.

### Neural Progenitor Cell Differentiation

Neural EBs (200 mESCs per drop) were hung for 2 days before transferring to ultra-low attachment 96-well plate and EBs were further cultivated for 3 days. One EB was placed in one well so that size and morphology could be examined under a microscope. Day 5 EBs were collected and placed into a 24-well cell culture plate for attachment. After 1 day, NSC selection was started by switching the medium to ITSFn medium, followed by NSC expansion in the N2 medium. This corresponds to Stage 3 and Stage 4 of the 5-stage neural differentiation method described above.

### Cardiac Progenitor Differentiation

Cardiac EBs (500 mESCs per drop) were hung for 3 days before transferring to a 60 mm bacteriological Petri dish and cultured for 2 days in suspension. Day 5 EBs were placed in gelatin-coated 6-well plates (about 15 EBs per well) for attachment and differentiation in Differentiation Medium I. This was sufficient to drive cardiac differentiation as beating EBs were observed from both R1 and HN after 7 days. The medium was changed every 2 days for 2 weeks (Boheler et al., 2002; Schroeder et al., 2009).

### Pancreatic Progenitor Differentiation

Pancreatic EBs (600 mESCs per drop) were hung for 3 days before transferring to a 60 mm bacteriological Petri dish and cultured for 2 days in suspension. Day 5 EBs were plated on gelatin-coated 6-well plates for attachment and differentiation in Differentiation Medium I. The medium was changed every 2 days for 9 days. Cells were trypsinized and seeded onto poly-L-ornithine/laminin-coated plates in the pancreatic differentiation medium. The medium was composed of DMEM/F12 supplemented with 1X P/S/Q, 25 μg/ml insulin, 50 μg/ml human apo-transferrin, 30 nM selenium chloride, 20 nM progesterone, 100 μM putrescine, 1 μg/ml laminin, 2% B27 and 10 mM nicotinamide. The medium was changed every 2 days for 3 weeks (Blyszczuk et al., 2004; Schroeder et al., 2009).

In lineage differentiation experiments, cell morphology was monitored under a microscope throughout the differentiation protocol. Cells at different stages were collected for RNA extraction and progenitor-specific markers were detected by RT-PCR.

### RNA Extraction, cDNA Synthesis, RT-PCR

Total RNA was extracted using TRI-Reagent (Sigma-Aldrich) followed by DNase-I treatment (Promega). For each sample, 1 μg of total RNA was used to synthesize cDNA using the First-Strand cDNA Synthesis Kit and 10X primer mix from USB (Affymetrix). qPCR was carried out on equal volumes of RT reactions using the 2X SYBR^®^ Green PCR Master Mix (Applied Biosystems). Cycling parameters used were: denaturation at 95°C for 30 s, annealing at 52–60°C for 40 s depending on primers, and elongation at 72°C for 45 s, for 45 cycles. qPCR data was calculated after normalization to GAPDH or 18S ribosomal RNA. Primer sequences are provided in Supplementary Table 1.

### Western Blot Analysis

Mouse embryonic stem cells were lysed in total lysis buffer [20 mM Tris-HCl (pH 8.0), 137 mM NaCl, 1 mM EDTA, 1% Triton X-100, 10% glycerol, 1.5 mM MgCl_2_, 1 mM DTT, 1 mM PMSF, and 1X protease inhibitor cocktail] and cell lysate extracted by centrifugation at 13,000 rpm for 30 min at 4°C. Protein concentrations were measured by Bradford assay (Bio-Rad). Fifty micrograms of total protein was loaded in each lane of an SDS-polyacrylamide gel.

Cytoplasmic and nuclear fractions were extracted using NE-PER Nuclear and Cytoplasmic Extraction Reagents (Pierce Biotechnology). From 20 μl of packed cells as starting material, about 200 μl of cytoplasmic fraction and 100 μl of nuclear fraction were obtained. Five percentage of total volume was loaded in each lane.

The following antibodies were used at the indicated dilution: rabbit α-Kdm6A (1:2000, Bethyl Laboratories), rabbit α-Olig2 (1:1000, Novus Biologicals), rabbit a-Vinculin (1:4000, Thermo Fisher Scientific), rabbit α-YY1 (1:2000, Santa Cruz Biotechnology), IRDye 680RD goat α-rabbit secondary antibody (1:10000, LI-COR Biosciences). Fluorescent signal was scanned using the LI-COR Odyssey machine.

### Statistical Analysis

Unless stated otherwise, values in the figures and text are presented as mean ± SEM. Statistical analysis was performed using Prism 7 (GraphPad Software). One-way analysis of variance (ANOVA) was used followed by Bartlett’s test. ^∗∗∗∗^*P* < 0.0001 represents statistical significance.

### RNA-Seq

Total RNA was isolated from three different batches of R1 and HN mESCs collected on different dates using the Direct-zol^TM^ RNA MiniPrep Kit (Zymo Research) according to the manufacturer’s instructions. Bioanalyzer was used to determine RNA concentration and quality. One hundred and thirty nanograms of total RNA was used to construct RNA-Seq libraries using Illumina TruSeq RNA Library Preparation Kit v2 and 15 cycles of PCR amplification. Libraries were run on an Illumina HiSeq 2500 instrument using a paired end 50 protocol, rapid run flow cell.

Sequencing results were demultiplexed and converted to FASTQ format using Illumina bcl2fastq software. Reads were aligned to the mouse genome (build mm10/GRCm38) using the splice-aware STAR aligner (Dobin et al., 2013). PCR duplicates were removed using the Picard toolkit^1^. HTSeq package (Anders et al., 2015) was utilized to generate counts for each gene based on how many aligned reads overlap its exons. These counts were then normalized and used to test for differential expression using negative binomial generalized linear models implemented by the DESeq2 R package (Love et al., 2014). Additionally, differential exon usage analysis was performed using the DEXSeq R package (Anders et al., 2012). The RNA-seq data has been deposited to the NCBI GEO database with an accession number GSE92905.

## Results

### mESCs Lacking Htt Fail to Differentiate into Neural Stem Cells

**FIGURE 1:**
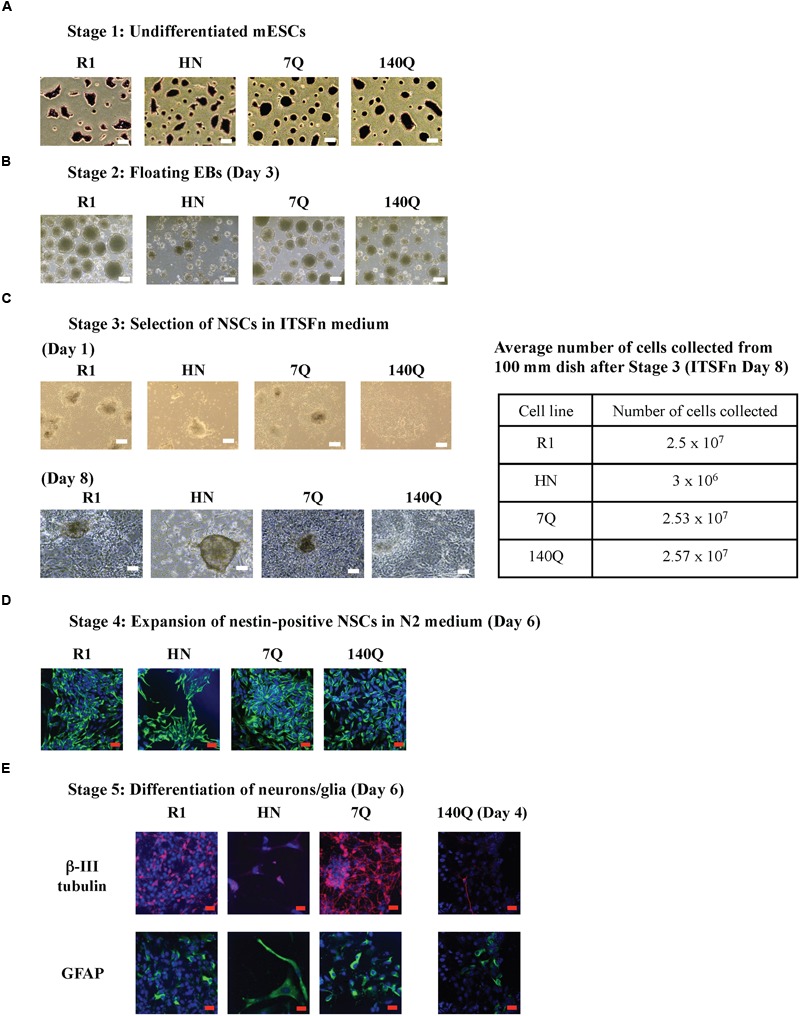
**Neural differentiation of mESC lines carrying different forms of Htt.** Using the 5-stage neural differentiation method, four mESC lines were differentiated into neurons/glia. Morphology of cells at different stages is shown. **(A)** Stage 1: mESCs. The pluripotency of mESCs was examined by the AP assay. **(B)** Stage 2: floating EBs at Day 3. **(C)** Stage 3: Selection of NSCs in ITSFn medium. EBs were plated onto cell culture plates for attachment and differentiation. Morphology of NSCs on Day 1 and Day 8 are shown. The average number of cells collected from one 100 mm dish after Stage 3 (ITSFn, Day 8) for each cell line is shown in the table. **(D)** Stage 4: Expansion of nestin-positive NSCs in N2 medium at Day 6. Anti-nestin (green) and TO-PRO-3 dye (blue, nuclei). **(E)** Stage 5: Differentiation of neurons/glia at Day 6 (R1, HN, 7Q) and Day 4 (140Q). Shown are β-III tubulin in red, GFAP in green, and TO-PRO-3 in blue. Scale bar: **(A–C)** 200 μm, **(D,E)** 25 μm.

We investigated potential role for wild type and mutant Htt in the differentiation of mESCs to cells of neural lineage. The four mESC lines tested were: wild type R1 ES cells, *Htt* nullizygous HN ES cells (Zeitlin et al., 1995), 7Q ES cells that express Flag-tagged wild type Htt (3xFlag-Htt^7Q/7Q^), heterozygous 140Q ES cells carrying an allele encoding Flag-tagged expanded polyQ Htt (3xFlag-Htt^140Q/7Q^) (Zheng et al., 2012). Expression of Htt and pluripotency markers was verified by immunoblotting and RT-qPCR (Supplementary Figure 1). The R1, HN, 7Q, and 140Q ES cells were differentiated into neurons/glia using the 5-stage neural differentiation method involving generation of massive EBs (Okabe et al., 1996; Lee et al., 2000). mESCs (Stage 1) were cultured under feeder-free conditions and undifferentiated status confirmed by dark purple staining of ES colonies in the AP assay (**Figure 1A**). Floating EBs (Stage 2) were spontaneously generated after seeding mESCs to a non-adherent Petri dish. All four mESCs formed EBs of different sizes. HN mESCs formed EBs much smaller than other three lines (**Figure 1B**). After 4 days in suspension, EBs were transferred to cell culture plates for attachment and differentiation. This was the start of Stage 3, selection of nestin-positive NSCs. After 1 day in the ITSFn medium, all EBs from R1, 7Q, and 140Q lines attached and started to differentiate. However, less than half of HN EBs attached and showed little signs of differentiation (**Figure 1C**). Cells were cultured in ITSFn for seven more days. R1, 7Q, and 140Q lines continued to differentiate and NSCs spread across the entire culture plate (**Figure 1C**, Day 8). After several rounds of changes of the ITSFn medium over 8 days, only a low percentage of HN EBs remained attached, with limited differentiation. Nestin-positive NSCs (Stage 4) were expanded by collecting and replating the Day 8 ITSFn cells into N2 medium. On average, nearly 10-fold less cells were obtained from HN (**Figure 1C**; Table). After culturing for 6 days in the N2 medium, cells were stained for an NSC marker nestin. Nearly all differentiated cells expressed nestin (**Figure 1D**), indicating successful generation of NSCs. They were next differentiated into neurons/glia (Stage 5). A mixture of neurons (β-III tubulin-positive) and glia (GFAP-positive) was obtained from both R1 and 7Q lines (wild type for Htt, **Figure 1E**). Most of HN NSCs died leaving a few large cells stained positive for GFAP. For 140Q NSCs, cell death was observed during the final stage of differentiation (Stage 5, Day 4, **Figure 1E**; Supplementary Figure 2), consistent with a previous report (Conforti et al., 2013). In summary, cells with wild type or mutant Htt (R1, 7Q, and 140Q) were able to generate NSCs, but cells lacking Htt (HN) did not make the NSCs. Further, only wild type ES cells differentiated into neurons/glia.

### mESCs Lacking Htt Show Limited Capacity to Form Neural Progenitor Cells

The above results suggest a requirement for Htt in neural differentiation. We next employed differentiation protocols involving hanging-drop EB formation. A defined number of mESCs was used to form one EB so that all EBs generated would be of similar size (Schroeder et al., 2009). These protocols were used to produce progenitors of ectoderm, endoderm, and mesoderm lineages.

**FIGURE 2:**
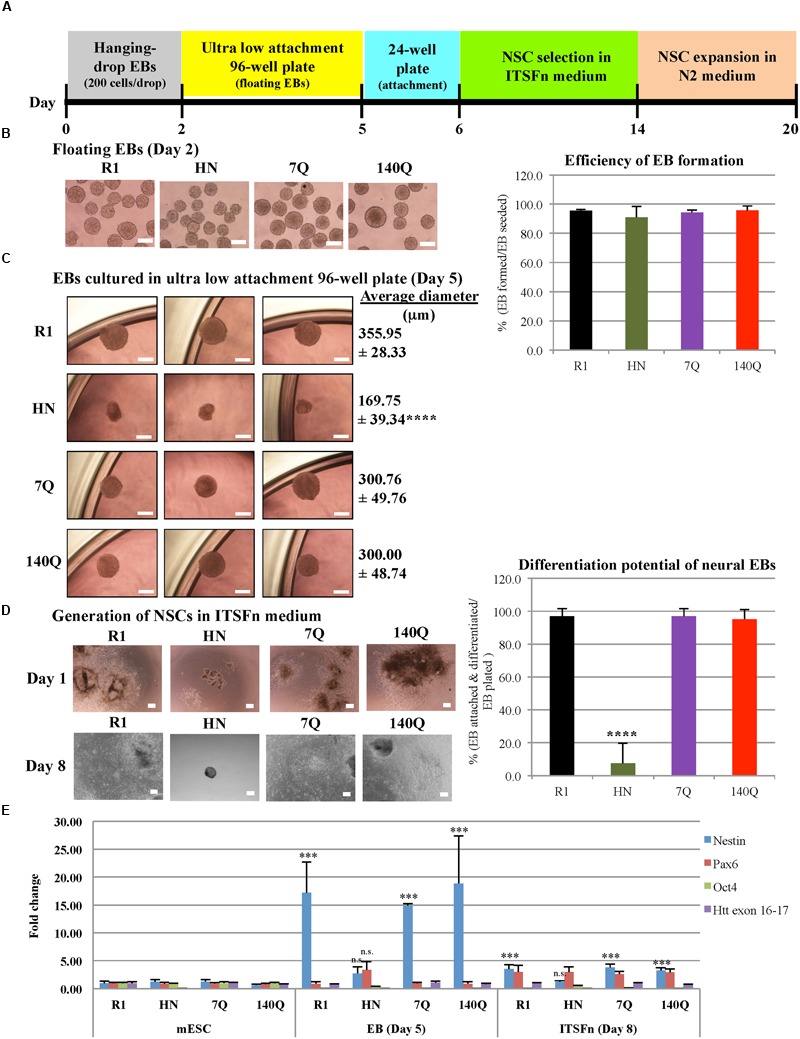
**Neural progenitor cell differentiation by the hanging-drop EB method (ectodermal lineage). (A)** Diagram showing the timeline of neural progenitor differentiation. **(B)** Morphology of floating EBs at Day 2. Graph at right shows efficiency of EB formation, calculated as the percentage of EBs formed over the total number of EBs seeded. R1: 95.62 ± 0.75, HN: 91.04 ± 7.41, 7Q: 94.37 ± 1.71, 140Q: 95.88 ± 2.88. No statistical significance. **(C)** Morphology and average diameter of Day 5 EBs cultured in ultra low attachment 96-well plate. ^∗∗∗∗^*P* < 0.0001, compared to R1, 7Q, and 140Q, by one-way ANOVA test. *n* = 3 independent experiments. **(D)** Generation of NSCs. Morphology of NSCs cultured in ITSFn medium at Day 1 and Day 8. Differentiation potential of neural EBs was calculated as the percentage of EBs attached and differentiated over the total number of EBs plated. R1: 97.09 ± 4.47, HN: 7.60 ± 12.04, 7Q: 97.08 ± 4.76, 140Q: 95.31 ± 5.86. ^∗∗∗∗^*P* < 0.0001, compared to R1, 7Q, and 140Q, by one-way ANOVA test. *n* = 3 independent experiments. Scale bar **(B–D)**: 200 μm. **(E)** Expression of different markers during neural progenitor cell differentiation. Quantitative RT-qPCR was performed for Nestin, Pax6, Oct4, Htt exon 16-17, and GAPDH using RNA collected from cells at different neural differentiation stages including mESC, EB (Day 5), and ITSFn (Day 8). Gene expression was calculated as fold change (over R1 mESC) after normalization to GAPDH expression. ^∗∗∗^*P* < 0.001, compared to mESCs, by Dunnett’s multiple comparisons test (one-way ANOVA). n.s., non-significant (*P* > 0.05). Separate graphs for Oct4 and Htt exon 16-17 are shown in Supplementary Figure 3A.

Neural progenitor cell differentiation (ectodermal lineage) was carried out in four mESC lines (**Figure 2A**). All made intact EBs (spheres); however, on average HN neural EBs were smaller in size (**Figure 2B**). Instead of using 60 mm Petri dishes, we modified the original protocol by transferring day 2 neural EBs to ultra low attachment 96-well plates. This way we could monitor floating EBs one by one under a microscope. For each EB, it was counted as ‘formed’ if it had a good spherical structure. The efficiency of EB formation, calculated as the number of EBs formed over EBs seeded, was similar for all four cell lines (around 95%, **Figure 2B**). After culturing in ultra low attachment 96-well plates for 3 days, bright field images of Day 5 neural EBs were captured. All EBs generated from R1, 7Q, and 140Q lines remained spherical, with an average diameter above 300 μm (**Figure 2C**). In contrast, HN neural EBs were much smaller (50% of R1 neural EBs) and appeared disorganized in structure. Day 5 neural EBs were transferred to cell culture plates for attachment and differentiation to NSCs in ITSFn medium. On Day 1 in the ITSFn medium, almost all R1, 7Q, and 140Q neural EBs attached to cell culture plates and started to differentiate. However, most HN neural EBs did not attach. Attached cells if any did not differentiate. Differentiation potential of neural EBs was calculated as the percentage of EBs attached and differentiated over the total number of EBs plated. HN neural EBs showed low efficiency of differentiation (<10%), while R1, 7Q, and 140Q neural EBs efficiently generated NSCs (>95%) (**Figure 2D**). NSC selection in ITSFn medium was carried out for 8 days. Only a few HN neural EBs attached to cell culture plates with no sign of differentiation (**Figure 2D**, Day 8). In contrast, neural EBs of R1, 7Q, and 140Q cell lines differentiated into NSCs and occupied nearly the entire plate.

Quantitative RT-qPCR was performed for Nestin, Pax6, Oct4, Htt exon 16-17, and GAPDH using RNA collected from cells at different neural differentiation stages (**Figure 2E**; Supplementary Figure 3A). We found expression of Nestin elevated in R1, 7Q, and 140Q EBs (Day 5) and ITSFn NSCs (Day 8), but not in HN cells. Lack of Nestin induction in HN ITSFn NSCs agreed with the observation that HN was less efficient in generating NSCs compared to stem cells expressing wild type or mutant Htt. We also examined Pax6 levels during neural differentiation. In ITSFn NSCs, all four cell lines showed elevated Pax6, however, they were not statistically significant. At the mESC stage, all four cell lines showed expression of the pluripotent gene Oct4 (Supplementary Figure 3A). Upon neural differentiation to EBs and ITSFn NSCs, Oct4 mRNA levels were dramatically reduced to less than 10% for R1, 7Q, and 140Q cells. By contrast HN cells maintained relatively high Oct4 expression in EBs (34%) and ITSFn NSCs (50%). This is consistent with the observation that HN EBs did not readily attach and differentiate into NSCs when floating EBs were placed in cell culture plates. As expected Htt (exon 16-17) expression in HN cells was 10-fold less than R1, 7Q, and 140Q cells at all stages of differentiation. The results suggest that HN mESCs have limited capacity in generating neural progenitor cells.

### mESCs Lacking Htt Can Differentiate into Pancreatic and Cardiac Progenitor Cells

**FIGURE 3:**
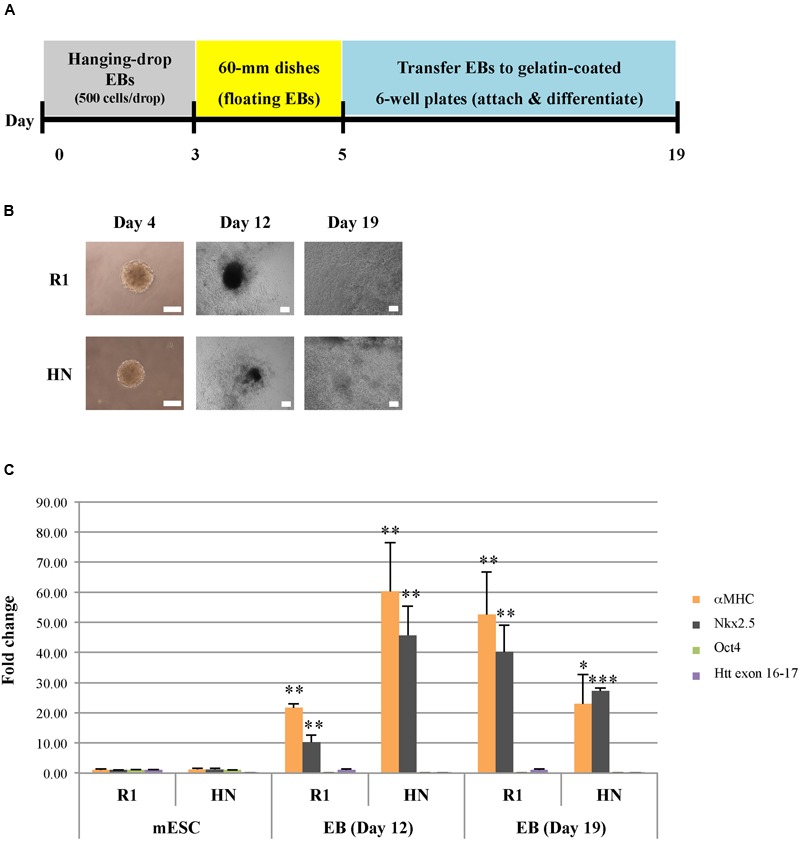
**Cardiac progenitor cell differentiation by the hanging-drop EB method (mesodermal lineage). (A)** Diagram showing the timeline of cardiac progenitor differentiation. **(B)** Morphology of R1 and HN cells during cardiac progenitor differentiation. Scale bar: 200 μm. **(C)** mRNA expression of cardiac progenitor cell-specific markers αMHC and Nkx2.5 at Day 12 and 19 was determined by quantitative RT-qPCR. Gene expression was calculated as fold change (over R1 mESC) after normalization to GAPDH expression. ^∗∗∗^*P* < 0.001, ^∗∗^*P* < 0.01, and ^∗^*P* < 0.05, compared to the mESC group by Dunnett’s multiple comparisons test (one-way ANOVA). Separate graphs for Oct4 and Htt exon 16-17 are shown in Supplementary Figure 3B.

We next sought to determine whether Htt is also required for endoderm and mesoderm differentiation. Cardiac progenitor differentiation (mesodermal lineage) was carried out on R1 and HN mESCs using the hanging-drop EB method, with 500 mES cells in each droplet (**Figure 3A**). At Day 4, cardiac EBs remained intact in both cell lines, although the size of HN cardiac EBs was smaller (**Figure 3B**). On Day 5, cardiac EBs were transferred to cell culture plates. Almost all EBs attached and differentiated over the following 2 weeks. Both R1 and HN mESCs differentiated into cardiac progenitor cells with similar morphology on Day 12 and Day 19 (**Figure 3B**). RT-qPCR analysis showed presence of cardiac progenitor-specific markers Nkx2.5 and αMHC in differentiated cells (**Figure 3C**). Interestingly, we found expression of the markers to be greater at Day 12 in HN cardiac progenitors compared with Day 19 cells, while R1 cells showed induction of these two markers as mESCs differentiated to Day 12 and Day 19 cardiac EBs. The observed difference suggests Htt may play a limited role in this process. At the mESC stage, both R1 and HN cells showed expression of the pluripotent gene Oct4 (Supplementary Figure 3B). Upon cardiac progenitor cell differentiation, Oct4 mRNA levels were greatly reduced to about 10% or less in both cell lines. Immunocytochemistry of Desmin, a specific marker expressed in Day 11 cardiac EBs, showed positive staining in both R1 and HN Day 12 cardiac EBs (Supplementary Figure 4). The results suggest that *Htt* is not required for cardiac progenitor cell differentiation under the experimental conditions used in this study.

**FIGURE 4:**
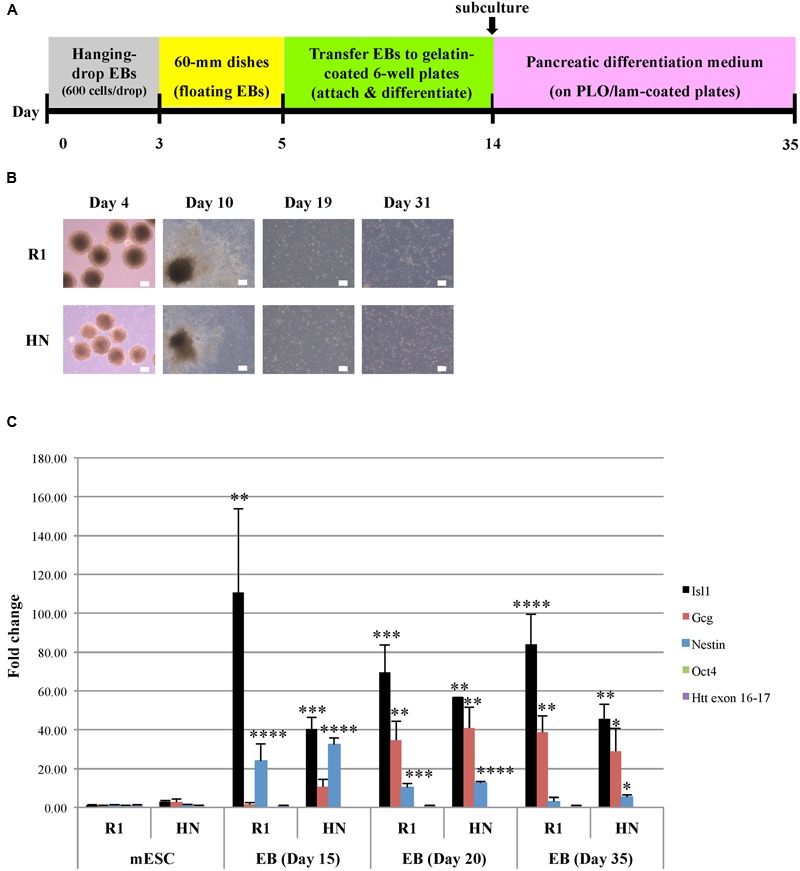
**Pancreatic progenitor cell differentiation by the hanging-drop EB method (endodermal lineage). (A)** Diagram showing the timeline of pancreatic progenitor differentiation. **(B)** Morphology of R1 and HN cells during pancreatic progenitor differentiation. Scale bar: 200 μm. **(C)** mRNA expression of pancreatic progenitor cell-specific marker Isl1 and Gcg at Day 15, 20, and 35 was determined by quantitative RT-qPCR. Gene expression was calculated as fold change (over R1 mESC) after normalization to GAPDH expression. ^∗∗∗∗^*P* < 0.0001, ^∗∗∗^*P* < 0.001, ^∗∗^*P* < 0.01, and ^∗^*P* < 0.05, compared to the mESC group by Dunnett’s multiple comparisons test (one-way ANOVA). Separate graphs for Oct4 and Htt exon 16-17 are shown in Supplementary Figure 3C.

Next, we carried out differentiation of R1 and HN mESCs into pancreatic progenitor cells (endodermal lineage) (**Figure 4A**). Similar to cardiac progenitor differentiation, pancreatic EBs formed from both cell lines, albeit those from HN EBs were smaller in size (**Figure 4B**). We found almost all pancreatic EBs attach and differentiate after plating Day 5 EBs onto cell culture plates. Similar morphologies were observed between R1 and HN differentiated cells (**Figure 4B**, Day 10). On Day 14, differentiated cells were collected and replated for further differentiation into pancreatic progenitors. Both R1 and HN cell lines gave rise to morphologically similar pancreatic progenitors as shown for Day 19 and Day 31 (**Figure 4B**). Quantitative RT-PCR analysis demonstrated mRNA expression of Isl1, a marker of pancreatic progenitors, in both R1 and HN differentiated cells at Day 20 and Day 35 (**Figure 4C**). Gcg (glucagon) is a pancreatic hormone whose expression appears at later stages of pancreatic progenitor cell differentiation. RT-qPCR data showed induction of Gcg in both R1 and HN pancreatic EBs after 20 days of differentiation. At Day 15, we observed differences in the levels of Isl1 and Gcg expression between R1 and HN cells, indicating the absence of Htt may have an effect on early stages of pancreatic differentiation. Nestin is also a marker during pancreatic differentiation. Its expression was found elevated dramatically in both R1 and HN EBs on Day 15 (>20-fold). Nestin levels decreased gradually as pancreatic differentiation continued. At the mESC stage, both R1 and HN cells showed expression of the pluripotent gene Oct4 (Supplementary Figure 3C). Upon pancreatic progenitor cell differentiation, Oct4 mRNA levels were greatly reduced to about 10% in both cell lines. The results suggest that *Htt* is also not required for pancreatic progenitor cell differentiation under the experimental conditions used in this study.

In these experiments, 500 and 600 mES cells per droplet were used to generate cardiac and pancreatic EBs, respectively. Since neural EBs were made with 200 mESCs per droplet we repeated the experiment with 500 and 1000 mES cells per droplet (Supplementary Figure 5). We observed HN Day 5 neural EBs slightly larger when the cell number increased from 500 to 1000 per droplet. However, they remained disorganized similar to HN neural EBs that were generated from 200 cells per droplet. Further differentiation in ITSFn medium (Day 1 and Day 6) showed that HN neural EBs from 500 or 1000 cells per droplet did not attach and differentiate into NSCs efficiently. Therefore, increasing the starting number of mESCs to generate neural EBs did not alter the poor efficiency of *Htt*-null cells in generating NSCs.

### Transcriptional Profiles of R1 and HN mESCs by RNA-Seq

The neural differentiation experiments showed inefficient generation of NSCs from HN mESCs, suggesting *Htt* plays a critical role in this process. To investigate the basis for the lack of neural differentiation we carried out gene expression profiling by RNA-seq to examine if genes involved in neural differentiation were dysregulated in HN mESCs.

**FIGURE 5:**
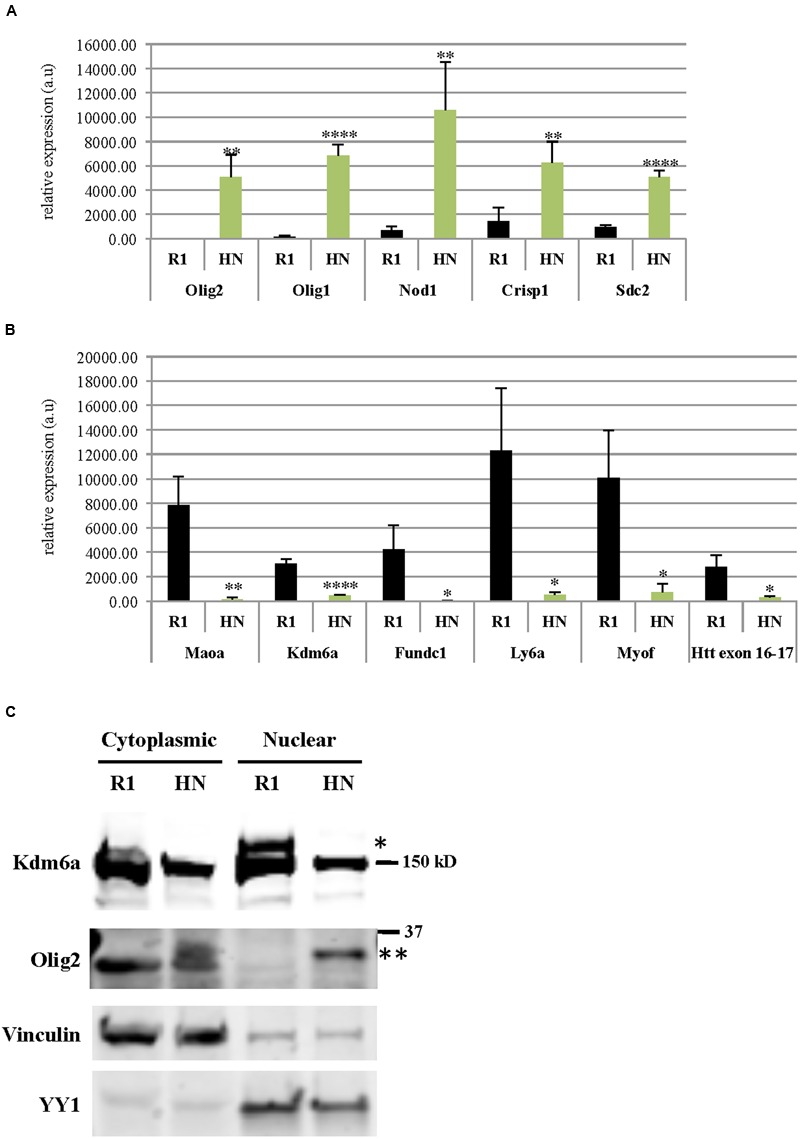
**Differentially expressed genes in R1 and *Htt*-null mESCs. mRNA levels of genes (A)** up-regulated or **(B)** down-regulated in HN mESCs compared with R1 cells were confirmed by quantitative RT-qPCR. Gene expression was calculated as relative expression after normalization to18S rRNA. All experiments were carried out using RNA samples extracted from four different batches of R1 and HN mESCs (*N* = 4). ^∗∗∗∗^*P* < 0.0001, ^∗∗^*P* < 0.01, and ^∗^*P* < 0.05, compared to R1, by unpaired *t*-test. a.u., arbitrary units. **(C)** Protein levels of Olig2 and Kdm6a were examined by immunoblotting. Vinculin and YY1 served as markers of cytoplasmic and nuclear fractions, respectively. ^∗^Kdm6a (170 kD), ^∗∗^Olig2 (32 kD).

Using RNA isolated from undifferentiated R1 and HN mESCs, we performed 50 nucleotide paired-end total mRNA sequencing. Comparison of expression profiles between HN and R1 mESCs identified 51 up-regulated genes (log2 fold change > 1, *p* < 0.05) and 48 down-regulated genes (log2 fold change < -1, *p* < 0.05). **Tables 1** and **2** show top 20 up-regulated and down-regulated genes in HN mESCs. Validation of the RNA-seq data carried out by RT-qPCR demonstrated elevated expression of Olig2, Olig1, Nod1, Crisp1, and Sdc2 in HN mESCs (**Figure 5A**), while Maoa, Kdm6a, Fundc1, Ly6a, and Myof mRNA levels were found reduced in HN mESCs (**Figure 5B**). Immunoblotting confirmed increased levels of Olig2 protein and reduced levels of Kdm6a protein in the nucleus (**Figure 5C**).

**Table 1:** Top 20 up-regulated genes in *Htt*-null mESCs (HN compared with R1).

Gene symbol	Gene description	log2 fold change	*p*-value	*p*-adj
Alg13	Asparagine-linked glycosylation 13 homolog (S. cerevisiae)	2.621	6.84E-28	2.40E-24
Galnt6	UDP-*N*-acetyl-alpha-D-galactosamine:polypeptide *N*-acetylgalactosaminyltransferase 6	2.133	4.89E-12	4.03E-09
Nod1	Nucleotide-binding oligomerization domain containing 1	2.120	1.30E-10	8.68E-08
Hist1h2bm	Histone cluster 1, H2bm	1.933	2.63E-28	1.85E-24
Olig1	Oligodendrocyte transcription factor 1	1.883	5.60E-09	3.02E-06
Snrpf	Small nuclear ribonucleoprotein polypeptide F	1.744	7.25E-18	1.02E-14
Mapt	Microtubule-associated protein tau	1.662	3.93E-12	3.44E-09
Sdc2	Syndecan 2	1.575	3.63E-08	1.64E-05
B3galtl	Beta-1,3-galactosyltransferase-like	1.555	1.57E-08	7.61E-06
Fam129a	Family with sequence similarity 129, member A	1.542	3.10E-10	1.98E-07
Ubd	Ubiquitin D	1.472	2.78E-05	5.57E-03
Uggt2	UDP-glucose glycoprotein glucosyltransferase 2	1.468	7.54E-06	1.85E-03
Cd68	CD68 molecule	1.460	7.16E-13	7.18E-10
Peg3	Paternally expressed 3	1.458	5.40E-06	1.43E-03
D2hgdh	D-2-hydroxyglutarate dehydrogenase	1.420	7.07E-06	1.77E-03
Fam25c	Family with sequence similarity 25, member C	1.412	7.13E-17	9.09E-14
Crisp1	Cysteine-rich secretory protein 1	1.318	1.83E-04	2.54E-02
Tgds	TDP-glucose 4,6-dehydratase	1.294	1.28E-05	2.85E-03
Olig2	Oligodendrocyte transcription factor 2	1.281	2.69E-04	3.52E-02
Trim34a	Tripartite motif containing 34A	1.253	3.76E-04	4.59E-02

**Table 2:** Top 20 down-regulated genes in *Htt*-null mESCs (HN compared with R1).

Gene symbol	Gene description	log2 fold change	*p*-value	*p*-adj
Maoa	Monoamine oxidase A	-4.129	6.59E-45	9.25E-41
Kdm6a	Lysine (K)-specific demethylase 6A	-3.481	5.22E-28	2.40E-24
Fundc1	FUN14 domain containing 1	-3.030	6.62E-21	1.03E-17
Hist1h4i	Histone cluster 1, H4i	-2.290	1.53E-23	3.07E-20
Tmem64	Transmembrane protein 64	-1.931	5.54E-25	1.30E-21
Khdc3	KH domain containing 3	-1.851	3.43E-11	2.53E-08
Htt	Huntingtin	-1.838	4.05E-10	2.47E-07
Myof	Myoferlin	-1.769	5.92E-10	3.46E-07
Hnf4a	Hepatocyte nuclear factor 4, alpha	-1.705	8.26E-07	2.47E-04
Wdfy1	WD repeat and FYVE domain containing 1	-1.625	5.75E-16	6.72E-13
Sfmbt2	Scm-like with four mbt domains 2	-1.607	1.88E-09	1.05E-06
Pttg1	Pituitary tumor-transforming 1	-1.580	6.37E-09	3.31E-06
Trh	Thyrotropin-releasing hormone	-1.568	1.08E-12	1.01E-09
Hist1h2bp	Histone cluster 1, H2bp	-1.537	5.50E-11	3.85E-08
Adam23	ADAM metallopeptidase domain 23	-1.508	7.97E-08	3.27E-05
Atp10d	ATPase, class V, type 10D	-1.489	1.94E-07	6.96E-05
Anxa3	Annexin A3	-1.399	1.11E-05	2.55E-03
Crxos1	Crx opposite strand transcript 1	-1.350	1.27E-04	1.94E-02
Eras	ES cell expressed Ras	-1.311	7.58E-08	3.22E-05
Fbxo2	F-box protein 2	-1.289	5.61E-05	9.59E-03

We used Gene Set Enrichment Analysis (Mootha et al., 2003; Subramanian et al., 2005) to compare up-regulated genes in HN mESCs with curated gene sets within the C2 collection of the Molecular Signatures Database (Subramanian et al., 2005). We found most enriched signature “Genes up-regulated in mouse ES cells with deficient SUZ12” (**Table 3**). SUZ12 is a component of Polycomb Repressive Complex 2 (PRC2). Interestingly, Olig2, reported repressed by PRC1 and PRC2 in mESCs (Boyer et al., 2006), is de-repressed in HN mESCs (**Figure 5A**; **Table 1**). Because there was a published report suggesting a link between Htt and components of the PRC2 complex (Seong et al., 2010), we asked whether Htt repressed the activation of Olig1 and Olig2 genes through the PRC2 complex. We performed chromatin immunoprecipitation analysis in R1 mESCs and failed to detect co-occupancy of Htt and Ezh2 (component of PRC2) at the Olig1 and Olig2 promoter regions (Supplementary Figure 6).

**Table 3:** Gene Set Enrichment Analysis finds ES cells with deficient SUZ12 share similar enriched signatures of up-regulated genes with *Htt*-null mESCs.

Gene Set Name	# Genes in Gene Set (K)			Description	# Genes in Overlap (k)	k/K	*p*-value	FDR *q*-value
PASINI_SUZ12_TARGETS_UP	112			Genes up-regulated in ES (embryonic stem cells) with deficient SUZ12 [GeneID = 23512].	15	0.1339	9.54E-18	4.51E-14



HTRA1	LAPTM5	NROB1	NFATC2IP	C17orf48
SPP1	LAMA1	H19	RPP25	CYCTP
TMEM40	PRDM14	AQP3	C17orf96	TEX15

*List of overlapping genes are shown above.*

## Discussion

### Phenotypic Differences between Wild Type and Mutant Htt in Neural Differentiation

In this study, we examined a role of Htt in neural differentiation *in vitro* using the 5-stage neural differentiation method in four mESC lines carrying different forms of Htt. Our data show that Htt is required for neural differentiation of mESCs. Htt is not needed for mesoderm or endoderm progenitor differentiation. RNA-seq analysis shows candidate dysregulated genes that may be involved in the inefficient NSC generation in mESCs lacking Htt.

Compared to wild type or mutant Htt-expressing cell lines (R1, 7Q, and 140Q), *Htt* nullizygous mESCs are not prone to generating NSCs as shown by small EB size and low tendency to attach to a plate and differentiate into NSCs. Further differentiation of HN NSCs to neurons/glia resulted in massive cell loss and formation of a few giant GFAP-positive cells. This observation is consistent with a recent report demonstrating that absence of Htt in mESCs promotes NSC differentiation into glial cell fate (Conforti et al., 2013). This conclusion is based on the increase of GFAP-positive cells at the terminal neuronal differentiation stage. In their report, both wild type and *Htt*-null ES cells generated similar numbers of NSCs. However, our data show that HN mESCs have limited capacity for generating NSCs. The discrepancy may be explained by different neural differentiation protocols used. Conforti et al. (2013) used a monolayer differentiation method (Spiliotopoulos et al., 2009), while we adopted the 5-stage neural differentiation protocol involving EB formation.

In contrast, neural EBs expressing mutant Htt (140Q) attached more readily to plates and differentiated into NSCs. Further differentiation of 140Q NSCs into neurons/glia resulted in cell death. These results agree with studies demonstrating that presence of polyQ-expanded Htt increases neural differentiation of embryonic and NSCs (Lorincz and Zawistowski, 2009), and cells with mutant polyQ Htt undergo cell death during neuronal differentiation (Conforti et al., 2013).

Studies by Nguyen et al. (2013b) found *Htt*-KO ESCs to be impaired for survival and specification of all three germ layers during EB formation and under inductive conditions, suggesting Htt is required for neural and non-neural differentiation. In contrast, mutant *Htt*-expressing cells showed alterations in stage-specific developmental events. The HN ESCs we examined were defective for differentiation into neural lineage, but not cardiac or pancreatic lineages. The *Htt*-KO ESCs used by above authors are deleted of exons 4 and 5 in the murine *Htt* gene (Duyao et al., 1995). The HN ESCs used in this study were created by deleting the promoter and exon 1 sequence of *Htt* (Zeitlin et al., 1995). Our 5-stage differentiation protocol also differed from the neural induction protocol used by Nguyen et al. (2013b). These differences may account for the different outcomes of ESCs undergoing differentiation. At a molecular level we observed differences in mRNA expression of cardiac and pancreatic progenitor markers between wild type and *Htt*-null cells. Thus, *Htt* appears to play a role in the pattern of gene expression through different stages of differentiation of cardiac and pancreatic progenitors.

### Transcriptional Profiling of mESCs Lacking *Htt*

The most down regulated gene in *Htt* null cells is MaoA, an enzyme that degrades amine neurotransmitters such as serotonin, dopamine, and noradrenalin. Its deficiency causes excess production of monoamine neurotransmitters resulting in enhanced aggressiveness in mice and humans (Brunner et al., 1993; Alia-Klein et al., 2008). Changes in the dopaminergic system have been correlated with HD pathogenesis (Chen et al., 2013; Schwab et al., 2015). Using well characterized immortalized mouse striatal cell lines ST*Hdh*^Q7/Q7^ and ST*Hdh*^Q111/Q111^, MaoA expression and activity were found elevated in cells expressing mutant Q111 Htt (Ooi et al., 2015). ST*Hdh*^Q111/Q111^ cells also showed enhanced susceptibility to stress and cell death. Interestingly, a recent study found polymorphisms in the *MAOA* gene as modifiers of psychiatric and cognitive symptoms in HD patients (Vinther-Jensen et al., 2016). MaoA may play a regulatory role in neural differentiation as mouse ES cells lacking *MaoA* show reduced differentiation to neural cells compared to wild type (Wang et al., 2011). Similar mechanisms may be responsible for the poor neural differentiation of *Htt*-null ES cells reported in this study.

Oligodendrocyte transcription factor 1 and 2 (Olig1 and Olig2) are basic helix-loop-helix transcription factors that promote oligodendrocyte specification in early neural progenitor cells. They were found up regulated in HN cells. It has been shown that Olig2 is repressed by polycomb repressive complexes in mESCs (Boyer et al., 2006). In a transgenic mouse model in which Olig2 was overexpressed in NSCs, severe defects in brain development and massive neuronal cell death of cortical progenitors were observed (Liu et al., 2015). Olig2 was shown to target enhancers of neurogenic genes and inhibit cortical neurogenesis. A recent mouse study revealed that Brg1, a subunit of the SWI/SNF chromatin remodeling complex, interacts with the Olig2 promoter and represses Olig2 expression in the cortex (Matsumoto et al., 2016). Although *Brg1*-null neural progenitor cells show early expression of Olig2 in the cortex, they fail to differentiate into oligodendrocyte progenitors. These studies suggest elevated levels of Olig2 in neural progenitor cells cause dysregulated neurogenesis or neuronal cell death.

A previous study examined gene expression profiles of mESCs with and without Htt (Strehlow et al., 2007). Microarray analysis showed diminished expression of vital patterning genes including *Lefty1, Otx2*, and *Pem* (*Rhox5*), as well as increased lysosomal activity genes such as *Casp8* and *Psmd8* in *Htt*-null mESCs (by at least twofold). We also found similar expression patterns of these genes in HN mESCs; however, fold changes were minimal (less than twofold). Although HN mESCs are the same in both studies, differences in gene expression profiling may be due to cell culture conditions and analytical methods used. We adopted the “3i + mLIF” formula to maintain the pluripotency of mESCs (Ying et al., 2008). Two additional studies reported RNA profiling of *Htt*-null cells by microarray (Jacobsen et al., 2011) and RNA-seq (Biagioli et al., 2015). We compared our RNA-seq data with the microarray study; however, we did not find any overlapping genes dysregulated by at least twofold. As mentioned above an important difference is that the cell line used in their study was the *Htt*-dKO ESCs (exon 4/5 deletion), while the HN ESCs used in our study were deleted of the promoter and exon 1 sequence. There may exist subtle molecular differences between the two cell lines.

We used Gene Set Enrichment Analysis to compare up-regulated genes in HN ESCs with curated gene sets in the Molecular Signatures Database. The most enriched signature was found in the up-regulated genes in mouse ES cells deficient of SUZ12, a component of the PRC2 repressor complex (**Table 3**). A link between PRC2 and HD has been reported. A recent genome-wide ChIP study for the H3K4me3 histone mark found altered enrichment in postmortem prefrontal cortex of HD patient samples compared with control brains (Dong et al., 2015). They also reported depletion of the PRC2 repressive state in H3K4me3-enriched peaks in HD, suggesting epigenetic modifications contribute to dysregulated gene expression associated with neurodegeneration. Further, PRC2 deficiency in striatal neurons resulted in transcriptional changes leading to neurodegeneration in mice (von Schimmelmann et al., 2016). Although PRC2 has been shown to be a direct target of Htt (Seong et al., 2010), we did not detect co-occupancy of Htt and Ezh2 (component of PRC2) at Olig1 and Olig2 promoter regions. Nevertheless, the finding that the transcriptional profile of *Htt*-null ES cells is most similar to that of cells lacking a PRC2 component lends further support to the idea that epigenetic gene regulation plays a role in HD pathogenesis.

## Author Contributions

MSY and NT conceived the study and designed the experiments, MSY performed the experiments and evaluated the data, MSY and NT wrote the paper.

## Conflict of Interest Statement

The authors declare that the research was conducted in the absence of any commercial or financial relationships that could be construed as a potential conflict of interest.

## Footnotes

1 http://broadinstitute.github.io/picard

## Abbreviations

EBs: embryoid bodies
GFAP: glial fibrillary acidic protein
HD: Huntington’s disease
Htt: huntingtin
ITSFn: insulin/transferrin/selenium/fibronectin
Kdm6a: lysine-specific demethylase 6A
MaoA: monoamine oxidase A
mESCs: mouse embryonic stem cells
NSCs: neural stem cells
PRC: polycomb repressive complex

## References

[B1] Alia-Klein N. Goldstein R. Z. Kriplani A. Logan J. Tomasi D. Williams B. (2008). Brain monoamine oxidase A activity predicts trait aggression. *J. Neurosci.* 28 5099–5104. 10.1523/JNEUROSCI.0925-08.2008 18463263 PMC2430409

[B2] Anders S. Pyl P. T. Huber W. (2015). HTSeq-a Python framework to work with high-throughput sequencing data. *Bioinformatics* 31 166–169. 10.1093/bioinformatics/btu638 25260700 PMC4287950

[B3] Anders S. Reyes A. Huber W. (2012). Detecting differential usage of exons from RNA-seq data. *Genome Res.* 22 2008–2017. 10.1101/gr.133744.111 22722343 PMC3460195

[B4] Arteaga-Bracho E. E. Gulinello M. Winchester M. L. Pichamoorthy N. Petronglo J. R. Zambrano A. D. (2016). Postnatal and adult consequences of loss of huntingtin during development: implications for Huntington’s disease. *Neurobiol. Dis.* 96 144–155. 10.1016/j.nbd.2016.09.006 27623015 PMC5102778

[B5] Batista C. M. Kippin T. E. Willaime-Morawek S. Shimabukuro M. K. Akamatsu W. van der Kooy D. (2006). A progressive and cell non-autonomous increase in striatal neural stem cells in the Huntington’s disease R6/2 mouse. *J. Neurosci.* 26 10452–10460. 10.1523/JNEUROSCI.2850-06.2006 17035529 PMC6674685

[B6] Biagioli M. Ferrari F. Mendenhall E. M. Zhang Y. Erdin S. Vijayvargia R. (2015). Htt CAG repeat expansion confers pleiotropic gains of mutant huntingtin function in chromatin regulation. *Hum. Mol. Genet.* 24 2442–2457. 10.1093/hmg/ddv006 25574027 PMC4383859

[B7] Blyszczuk P. Asbrand C. Rozzo A. Kania G. St-Onge L. Rupnik M. (2004). Embryonic stem cells differentiate into insulin-producing cells without selection of nestin-expressing cells. *Int. J. Dev. Biol.* 48 1095–1104. 10.1387/ijdb.041904pb 15602695

[B8] Boheler K. R. Czyz J. Tweedie D. Yang H. T. Anisimov S. V. Wobus A. M. (2002). Differentiation of pluripotent embryonic stem cells into cardiomyocytes. *Circ. Res.* 91 189–201. 12169644 10.1161/01.res.0000027865.61704.32

[B9] Boyer L. A. Plath K. Zeitlinger J. Brambrink T. Medeiros L. A. Lee T. I. (2006). Polycomb complexes repress developmental regulators in murine embryonic stem cells. *Nature* 441 349–353. 10.1038/nature04733 16625203

[B10] Brunner H. G. Nelen M. Breakefield X. O. Ropers H. H. van Oost B. A. (1993). Abnormal behavior associated with a point mutation in the structural gene for monoamine oxidase A. *Science* 262 578–580. 8211186 10.1126/science.8211186

[B11] Chen J. Y. Wang E. A. Cepeda C. Levine M. S. (2013). Dopamine imbalance in Huntington’s disease: a mechanism for the lack of behavioral flexibility. *Front. Neurosci.* 7:114 10.3389/fnins.2013.00114 PMC3701870 23847463

[B12] Conforti P. Camnasio S. Mutti C. Valenza M. Thompson M. Fossale E. (2013). Lack of huntingtin promotes neural stem cells differentiation into glial cells while neurons expressing huntingtin with expanded polyglutamine tracts undergo cell death. *Neurobiol. Dis.* 50 160–170. 10.1016/j.nbd.2012.10.015 23089356

[B13] Curtis M. A. Penney E. B. Pearson A. G. van Roon-Mom W. M. Butterworth N. J. Dragunow M. (2003). Increased cell proliferation and neurogenesis in the adult human Huntington’s disease brain. *Proc. Natl. Acad. Sci. U.S.A.* 100 9023–9027. 10.1073/pnas.1532244100 12853570 PMC166431

[B14] Dobin A. Davis C. A. Schlesinger F. Drenkow J. Zaleski C. Jha S. (2013). STAR: ultrafast universal RNA-seq aligner. *Bioinformatics* 29 15–21. 10.1093/bioinformatics/bts635 23104886 PMC3530905

[B15] Dong X. Tsuji J. Labadorf A. Roussos P. Chen J. F. Myers R. H. (2015). The role of H3K4me3 in transcriptional regulation is altered in Huntington’s disease. *PLoS ONE* 10:e0144398 10.1371/journal.pone.0144398 PMC4670094 26636336

[B16] Dunah A. W. Jeong H. Griffin A. Kim Y. M. Standaert D. G. Hersch S. M. (2002). Sp1 and TAFII130 transcriptional activity disrupted in early Huntington’s disease. *Science* 296 2238–2243. 10.1126/science.1072613 11988536

[B17] Duyao M. P. Auerbach A. B. Ryan A. Persichetti F. Barnes G. T. McNeil S. M. (1995). Inactivation of the mouse Huntington’s disease gene homolog Hdh. *Science* 269 407–410. 7618107 10.1126/science.7618107

[B18] Elias S. Thion M. S. Yu H. Sousa C. M. Lasgi C. Morin X. (2014). Huntingtin regulates mammary stem cell division and differentiation. *Stem Cell Reports* 2 491–506. 10.1016/j.stemcr.2014.02.011 24749073 PMC3986500

[B19] Gauthier L. R. Charrin B. C. Borrell-Pages M. Dompierre J. P. Rangone H. Cordelieres F. P. (2004). Huntingtin controls neurotrophic support and survival of neurons by enhancing BDNF vesicular transport along microtubules. *Cell* 118 127–138. 10.1016/j.cell.2004.06.018 15242649

[B20] Gil J. M. Mohapel P. Araujo I. M. Popovic N. Li J. Y. Brundin P. (2005). Reduced hippocampal neurogenesis in R6/2 transgenic Huntington’s disease mice. *Neurobiol. Dis.* 20 744–751. 10.1016/j.nbd.2005.05.006 15951191

[B21] Godin J. D. Colombo K. Molina-Calavita M. Keryer G. Zala D. Charrin B. C. (2010). Huntingtin is required for mitotic spindle orientation and mammalian neurogenesis. *Neuron* 67 392–406. 10.1016/j.neuron.2010.06.027 20696378

[B22] Jacobsen J. C. Gregory G. C. Woda J. M. Thompson M. N. Coser K. R. Murthy V. (2011). HD CAG-correlated gene expression changes support a simple dominant gain of function. *Hum. Mol. Genet.* 20 2846–2860. 10.1093/hmg/ddr195 21536587 PMC3118763

[B23] Kegel K. B. Kim M. Sapp E. McIntyre C. Castano J. G. Aronin N. (2000). Huntingtin expression stimulates endosomal-lysosomal activity, endosome tubulation, and autophagy. *J. Neurosci.* 20 7268–7278. 11007884 10.1523/JNEUROSCI.20-19-07268.2000 PMC6772788

[B24] Keryer G. Pineda J. R. Liot G. Kim J. Dietrich P. Benstaali C. (2011). Ciliogenesis is regulated by a huntingtin-HAP1-PCM1 pathway and is altered in Huntington disease. *J. Clin. Invest.* 121 4372–4382. 10.1172/JCI57552 21985783 PMC3223861

[B25] Leavitt B. R. Guttman J. A. Hodgson J. G. Kimel G. H. Singaraja R. Vogl A. W. (2001). Wild-type huntingtin reduces the cellular toxicity of mutant huntingtin in vivo. *Am. J. Hum. Genet.* 68 313–324. 10.1086/318207 11133364 PMC1235265

[B26] Leavitt B. R. van Raamsdonk J. M. Shehadeh J. Fernandes H. Murphy Z. Graham R. K. (2006). Wild-type huntingtin protects neurons from excitotoxicity. *J. Neurochem.* 96 1121–1129. 10.1111/j.1471-4159.2005.03605.x 16417581

[B27] Lee S. H. Lumelsky N. Studer L. Auerbach J. M. McKay R. D. (2000). Efficient generation of midbrain and hindbrain neurons from mouse embryonic stem cells. *Nat. Biotechnol.* 18 675–679. 10.1038/76536 10835609

[B28] Liu W. Zhou H. Liu L. Zhao C. Deng Y. Chen L. (2015). Disruption of neurogenesis and cortical development in transgenic mice misexpressing Olig2, a gene in the Down syndrome critical region. *Neurobiol. Dis.* 77 106–116. 10.1016/j.nbd.2015.02.021 25747816 PMC4428323

[B29] Lorincz M. T. Zawistowski V. A. (2009). Expanded CAG repeats in the murine Huntington’s disease gene increases neuronal differentiation of embryonic and neural stem cells. *Mol. Cell. Neurosci.* 40 1–13. 10.1016/j.mcn.2008.06.004 18625318 PMC2666278

[B30] Love M. I. Huber W. Anders S. (2014). Moderated estimation of fold change and dispersion for RNA-seq data with DESeq2. *Genome Biol.* 15 550 10.1186/s13059-014-0550-8 PMC4302049 25516281

[B31] Marcora E. Gowan K. Lee J. E. (2003). Stimulation of NeuroD activity by huntingtin and huntingtin-associated proteins HAP1 and MLK2. *Proc. Natl. Acad. Sci. U.S.A.* 100 9578–9583. 10.1073/pnas.1133382100 12881483 PMC170960

[B32] Matsumoto S. Banine F. Feistel K. Foster S. Xing R. Struve J. (2016). Brg1 directly regulates Olig2 transcription and is required for oligodendrocyte progenitor cell specification. *Dev. Biol.* 413 173–187. 10.1016/j.ydbio.2016.04.003 27067865 PMC4851915

[B33] Metzler M. Chen N. Helgason C. D. Graham R. K. Nichol K. McCutcheon K. (1999). Life without huntingtin: normal differentiation into functional neurons. *J. Neurochem.* 72 1009–1018. 10037472 10.1046/j.1471-4159.1999.0721009.x

[B34] Molero A. E. Arteaga-Bracho E. E. Chen C. H. Gulinello M. Winchester M. L. Pichamoorthy N. (2016). Selective expression of mutant huntingtin during development recapitulates characteristic features of Huntington’s disease. *Proc. Natl. Acad. Sci. U.S.A.* 113 5736–5741. 10.1073/pnas.1603871113 27140644 PMC4878495

[B35] Molero A. E. Gokhan S. Gonzalez S. Feig J. L. Alexandre L. C. Mehler M. F. (2009). Impairment of developmental stem cell-mediated striatal neurogenesis and pluripotency genes in a knock-in model of Huntington’s disease. *Proc. Natl. Acad. Sci. U.S.A.* 106 21900–21905. 10.1073/pnas.0912171106 19955426 PMC2799796

[B36] Molina-Calavita M. Barnat M. Elias S. Aparicio E. Piel M. Humbert S. (2014). Mutant huntingtin affects cortical progenitor cell division and development of the mouse neocortex. *J. Neurosci.* 34 10034–10040. 10.1523/JNEUROSCI.0715-14.2014 25057205 PMC6608303

[B37] Mootha V. K. Lindgren C. M. Eriksson K. F. Subramanian A. Sihag S. Lehar J. (2003). PGC-1alpha-responsive genes involved in oxidative phosphorylation are coordinately downregulated in human diabetes. *Nat. Genet.* 34 267–273. 10.1038/ng1180 12808457

[B38] Nasir J. Floresco S. B. O’Kusky J. R. Diewert V. M. Richman J. M. Zeisler J. (1995). Targeted disruption of the Huntington’s disease gene results in embryonic lethality and behavioral and morphological changes in heterozygotes. *Cell* 81 811–823. 7774020 10.1016/0092-8674(95)90542-1

[B39] Nguyen G. D. Gokhan S. Molero A. E. Mehler M. F. (2013a). Selective roles of normal and mutant huntingtin in neural induction and early neurogenesis. *PLoS ONE* 8:e64368 10.1371/journal.pone.0064368 PMC3653864 23691206

[B40] Nguyen G. D. Molero A. E. Gokhan S. Mehler M. F. (2013b). Functions of huntingtin in germ layer specification and organogenesis. *PLoS ONE* 8:e72698 10.1371/journal.pone.0072698 PMC3742581 23967334

[B41] Okabe S. Forsberg-Nilsson K. Spiro A. C. Segal M. McKay R. D. (1996). Development of neuronal precursor cells and functional postmitotic neurons from embryonic stem cells in vitro. *Mech. Dev.* 59 89–102. 8892235 10.1016/0925-4773(96)00572-2

[B42] Ooi J. Hayden M. R. Pouladi M. A. (2015). Inhibition of excessive monoamine oxidase A/B activity protects against stress-induced neuronal death in Huntington disease. *Mol. Neurobiol.* 52 1850–1861. 10.1007/s12035-014-8974-4 25398695 PMC4586002

[B43] Pal A. Severin F. Lommer B. Shevchenko A. Zerial M. (2006). Huntingtin-HAP40 complex is a novel Rab5 effector that regulates early endosome motility and is up-regulated in Huntington’s disease. *J. Cell Biol.* 172 605–618. 10.1083/jcb.200509091 16476778 PMC2063679

[B44] Ravikumar B. Vacher C. Berger Z. Davies J. E. Luo S. Oroz L. G. (2004). Inhibition of mTOR induces autophagy and reduces toxicity of polyglutamine expansions in fly and mouse models of Huntington disease. *Nat. Genet.* 36 585–595. 10.1038/ng1362 15146184

[B45] Rigamonti D. Bauer J. H. De-Fraja C. Conti L. Sipione S. Sciorati C. (2000). Wild-type huntingtin protects from apoptosis upstream of caspase-3. *J. Neurosci.* 20 3705–3713. 10804212 10.1523/JNEUROSCI.20-10-03705.2000 PMC6772672

[B46] Ritch J. J. Valencia A. Alexander J. Sapp E. Gatune L. Sangrey G. R. (2012). Multiple phenotypes in Huntington disease mouse neural stem cells. *Mol. Cell. Neurosci.* 50 70–81. 10.1016/j.mcn.2012.03.011 22508027 PMC3383872

[B47] Saudou F. Humbert S. (2016). The biology of Huntingtin. *Neuron* 89 910–926. 10.1016/j.neuron.2016.02.003 26938440

[B48] Schroeder I. S. Wiese C. Truong T. T. Rolletschek A. Wobus A. M. (2009). Differentiation analysis of pluripotent mouse embryonic stem (ES) cells in vitro. *Methods Mol. Biol.* 530 219–250. 10.1007/978-1-59745-471-1_12 19266345

[B49] Schwab L. C. Garas S. N. Drouin-Ouellet J. Mason S. L. Stott S. R. Barker R. A. (2015). Dopamine and Huntington’s disease. *Expert Rev. Neurother.* 15 445–458. 10.1586/14737175.2015.1025383 25773746

[B50] Seong I. S. Woda J. M. Song J. J. Lloret A. Abeyrathne P. D. Woo C. J. (2010). Huntingtin facilitates polycomb repressive complex 2. *Hum. Mol. Genet.* 19 573–583. 10.1093/hmg/ddp524 19933700 PMC2807366

[B51] Spiliotopoulos D. Goffredo D. Conti L. Di Febo F. Biella G. Toselli M. (2009). An optimized experimental strategy for efficient conversion of embryonic stem (ES)-derived mouse neural stem (NS) cells into a nearly homogeneous mature neuronal population. *Neurobiol. Dis.* 34 320–331. 10.1016/j.nbd.2009.02.007 19236914

[B52] Steffan J. S. Kazantsev A. Spasic-Boskovic O. Greenwald M. Zhu Y. Z. Gohler H. (2000). The Huntington’s disease protein interacts with p53 and CREB-binding protein and represses transcription. *Proc. Natl. Acad. Sci. U.S.A.* 97 6763–6768. 10.1073/pnas.100110097 10823891 PMC18731

[B53] Strehlow A. N. Li J. Z. Myers R. M. (2007). Wild-type huntingtin participates in protein trafficking between the Golgi and the extracellular space. *Hum. Mol. Genet.* 16 391–409. 10.1093/hmg/ddl467 17189290

[B54] Subramanian A. Tamayo P. Mootha V. K. Mukherjee S. Ebert B. L. Gillette M. A. (2005). Gene set enrichment analysis: a knowledge-based approach for interpreting genome-wide expression profiles. *Proc. Natl. Acad. Sci. U.S.A.* 102 15545–15550. 10.1073/pnas.0506580102 16199517 PMC1239896

[B55] Takano H. Gusella J. F. (2002). The predominantly HEAT-like motif structure of huntingtin and its association and coincident nuclear entry with dorsal, an NF-kB/Rel/dorsal family transcription factor. *BMC Neurosci.* 3:15 10.1186/1471-2202-3-15 PMC137586 12379151

[B56] Tattersfield A. S. Croon R. J. Liu Y. W. Kells A. P. Faull R. L. Connor B. (2004). Neurogenesis in the striatum of the quinolinic acid lesion model of Huntington’s disease. *Neuroscience* 127 319–332. 10.1016/j.neuroscience.2004.04.061 15262322

[B57] Twelvetrees A. E. Yuen E. Y. Arancibia-Carcamo I. L. MacAskill A. F. Rostaing P. Lumb M. J. (2010). Delivery of GABAARs to synapses is mediated by HAP1-KIF5 and disrupted by mutant huntingtin. *Neuron* 65 53–65. 10.1016/j.neuron.2009.12.007 20152113 PMC2841506

[B58] Vinther-Jensen T. Nielsen T. T. Budtz-Jorgensen E. Larsen I. U. Hansen M. M. Hasholt L. (2016). Psychiatric and cognitive symptoms in Huntington’s disease are modified by polymorphisms in catecholamine regulating enzyme genes. *Clin. Genet.* 89 320–327. 10.1111/cge.12628 26081309

[B59] von Schimmelmann M. Feinberg P. A. Sullivan J. M. Ku S. M. Badimon A. Duff M. K. (2016). Polycomb repressive complex 2 (PRC2) silences genes responsible for neurodegeneration. *Nat. Neurosci.* 19 1321–1330. 10.1038/nn.4360 27526204 PMC5088783

[B60] Wang Z. Q. Chen K. Ying Q. L. Li P. Shih J. C. (2011). Monoamine oxidase A regulates neural differentiation of murine embryonic stem cells. *J. Neural Transm.* 118 997–1001. 10.1007/s00702-011-0655-0 21607742 PMC3435112

[B61] White J. K. Auerbach W. Duyao M. P. Vonsattel J. P. Gusella J. F. Joyner A. L. (1997). Huntingtin is required for neurogenesis and is not impaired by the Huntington’s disease CAG expansion. *Nat. Genet.* 17 404–410. 10.1038/ng1297-404 9398841

[B62] Ying Q. L. Wray J. Nichols J. Batlle-Morera L. Doble B. Woodgett J. (2008). The ground state of embryonic stem cell self-renewal. *Nature* 453 519–523. 10.1038/nature06968 18497825 PMC5328678

[B63] Zala D. Hinckelmann M. V. Saudou F. (2013). Huntingtin’s function in axonal transport is conserved in *Drosophila melanogaster*. *PLoS ONE* 8:e60162 10.1371/journal.pone.0060162 PMC3610688 23555909

[B64] Zeitlin S. Liu J. P. Chapman D. L. Papaioannou V. E. Efstratiadis A. (1995). Increased apoptosis and early embryonic lethality in mice nullizygous for the Huntington’s disease gene homologue. *Nat. Genet.* 11 155–163. 10.1038/ng1095-155 7550343

[B65] Zheng S. Ghitani N. Blackburn J. S. Liu J. P. Zeitlin S. O. (2012). A series of N-terminal epitope tagged Hdh knock-in alleles expressing normal and mutant huntingtin: their application to understanding the effect of increasing the length of normal Huntingtin’s polyglutamine stretch on CAG140 mouse model pathogenesis. *Mol. Brain* 5:28 10.1186/1756-6606-5-28 PMC3499431 22892315

[B66] Zuccato C. Tartari M. Crotti A. Goffredo D. Valenza M. Conti L. (2003). Huntingtin interacts with REST/NRSF to modulate the transcription of NRSE-controlled neuronal genes. *Nat. Genet.* 35 76–83. 10.1038/ng1219 12881722

