# Supplementary material for: Huntingtin Is Required for Neural But Not Cardiac/Pancreatic Progenitor Differentiation of Mouse Embryonic Stem Cells In vitro
Source: Front Cell Neurosci. 2017 Feb 21;11:33. doi: 10.3389/fncel.2017.00033 (PMC5318384; doi:10.3389/fncel.2017.00033)
Supplement: Supplementary file 1 [file Table_1.PDF]

Supplementary Table 1: List of primers used for RT-PCR

Nestin Forward: CTACCAGGAGCGCGTGGC

Nestin Reverse: TCCACAGCCAGCTGGAACTT

18S rRNA Forward: AGGAATTGACGGAAGGGCACCA

18S rRNA Reverse: TTATCGGAATTAACCAGACAAATCG

Nkx2.5 Forward: CGACGGAAGCCACGCGTGCT

Nkx2.5 Reverse: CCGCTGTCGCTTGCACTTG

αMHC Forward: GGAAGAGCGAGCGGCGCATCAA

αMHC Reverse: CTGCTGGAGAGGTTATTCC

Isl1 Forward: GGTTGTACGGGATCAAATGC

Isl1 Reverse: ATGCTGCGTTTCTTGTCCTT

Olig2 Forward: GGCGGTGGCTTCAAGTCATC

Olig2 Reverse: TAGTTTCGCGCCAGCAGCAG

Olig1 Forward: TTCCGAGCTGGATGTTACGC

Olig1 Reverse: AGGGAAGTGGAGACTAAGTAAGG

Nod1 Forward: GAAGGCACCCCATTGGGTT

Nod1 Reverse: AATCTCTGCATCTTCGGCTGA

Crisp1 Forward: TGTCCTGTTGGCAATTATCAAGG

Crisp1 Reverse: CCACAACTATTGGTGCATAGCC

Sdc2 Forward: TGTGTCCGCAGAGACGAGAA

Sdc2 Reverse: GGAATCAGTTGGGATGTTGTCA

Maoa Forward: GCCCAGTATCACAGGCCAC

Maoa Reverse: CGGGCTTCCAGAACCAAGA

Kdm6a Forward: AATTGGGACCCTACTGGAAC

Kdm6a reverse: ACTCTCACGAAGGCAGGAAG

Fundc1 Forward: CCCCCTCCCCAAGACTATGAA

Fundc1 Reverse: CCACCCATTACAATCTGAGTAGC

Ly6a Forward: AGGAGGCAGCAGTTATTGTGG

Ly6a Reverse: CGTTGACCTTAGTACCCAGGA

Myof Forward: ACCGCTTTCGGTGTGATCC

Myof Reverse: GCCAGTAATGGTTTGGTGTCTTC
