# Supplementary material for: Huntingtin Is Required for Neural But Not Cardiac/Pancreatic Progenitor Differentiation of Mouse Embryonic Stem Cells In vitro
Source: Front Cell Neurosci. 2017 Feb 21;11:33. doi: 10.3389/fncel.2017.00033 (PMC5318384; doi:10.3389/fncel.2017.00033)

Supplementary Figure 1. (A) Htt proteins expression in different mESC lines. Total cell lysates were probed with α-Htt (Mab 2166), α-FLAG, and α-polyQ (3B5H10) antibodies. The blot probed for polyQ was stripped and re-probed for α-Vinculin as loading control. *Htt140Q; NS, non-specific. **(B)** Quantitative RT-qPCR analysis of pluripotent markers. Gene expression was calculated as fold change over R1 after normalization to GAPDH levels. n.s.; non-significant. **(C)** Oct4 protein expression is similar in all four cell lines as shown by immunoblotting. **(D)** Immunostaining of cell lines with α-Oct4 antibody indicates no obvious clonal differences. Scale bar: 25 µm

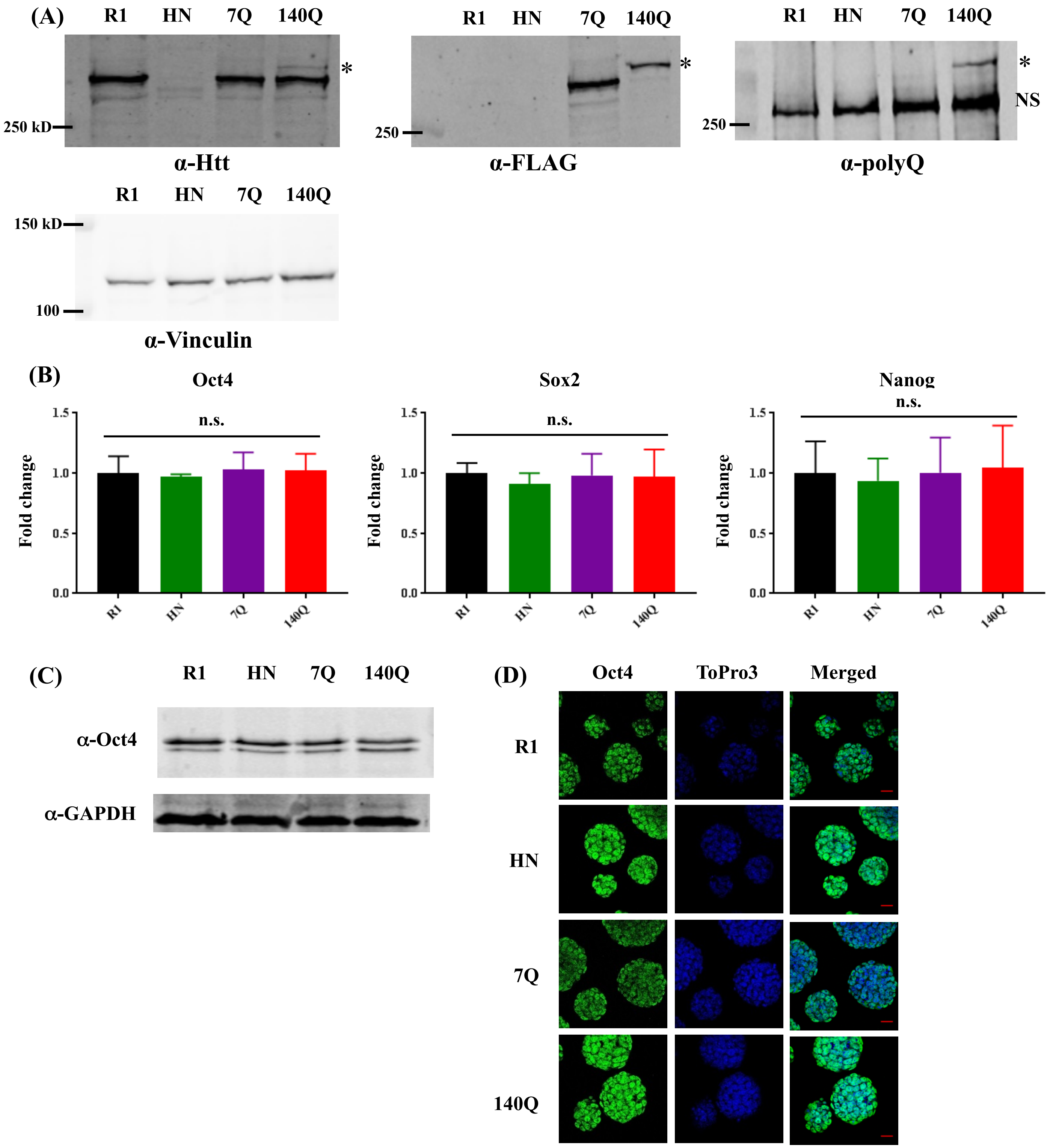

Supplement: Supplementary file 2 [file Image_1.PDF]
