# Supplementary material for: Huntingtin Is Required for Neural But Not Cardiac/Pancreatic Progenitor Differentiation of Mouse Embryonic Stem Cells In vitro
Source: Front Cell Neurosci. 2017 Feb 21;11:33. doi: 10.3389/fncel.2017.00033 (PMC5318384; doi:10.3389/fncel.2017.00033)

Supplementary Figure 2. Staining of β-III tubulin (red) and GFAP (green) in140Q Stage 5 neurons/glia at Day 4. (a) A relatively intact neuron (β -III tubulin positive) with some glia (GFAP positive). (b-e) Four different fields showing dot-like/broken-line neurites. (f) 2X zoom-out image of (e). Scale bar: 25 μm.

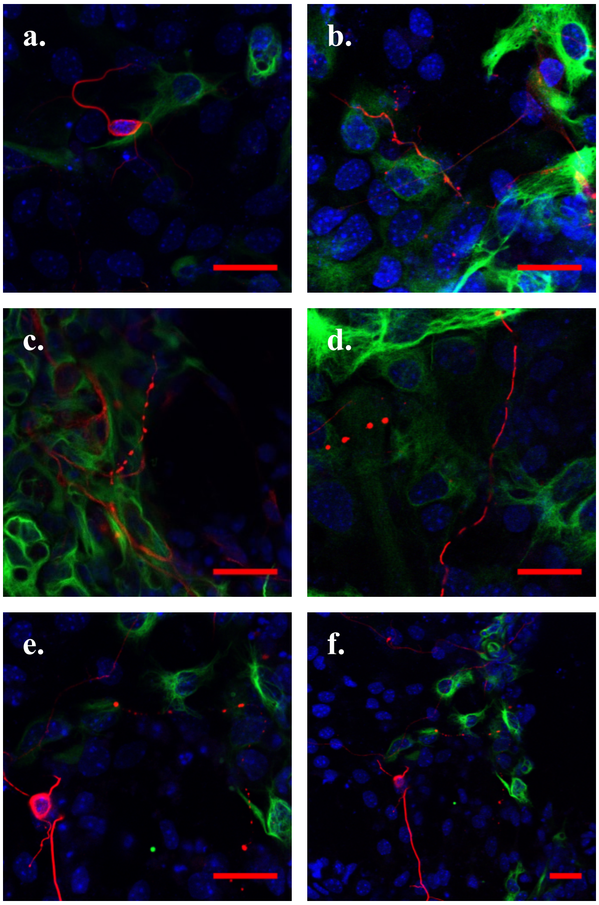

Supplement: Supplementary file 3 [file Image_2.PDF]
