# Supplementary material for: Huntingtin Is Required for Neural But Not Cardiac/Pancreatic Progenitor Differentiation of Mouse Embryonic Stem Cells In vitro
Source: Front Cell Neurosci. 2017 Feb 21;11:33. doi: 10.3389/fncel.2017.00033 (PMC5318384; doi:10.3389/fncel.2017.00033)

Supplementary Figure 3. Expression of Oct4 and Htt during (A) neural, (B) cardiac, and (C) pancreatic progenitor cell differentiation. RT-qPCR was performed using RNA collected at indicated progenitor cell differentiation stages. Gene expression was calculated as fold change (over R1 mESCs) after normalization to GAPDH expression. ****P< 0.0001, ***P< 0.001 compared to the mESC group by Dunnett's multiple comparisons test (one-way ANOVA).

(A)

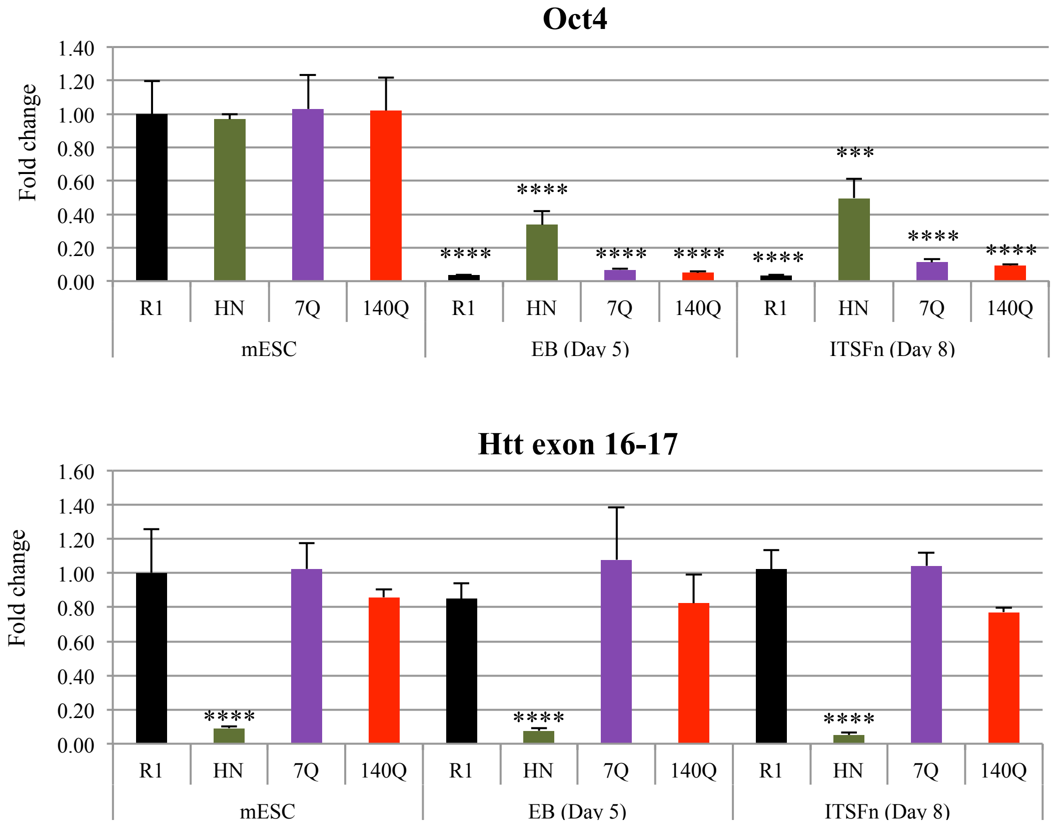

(B)

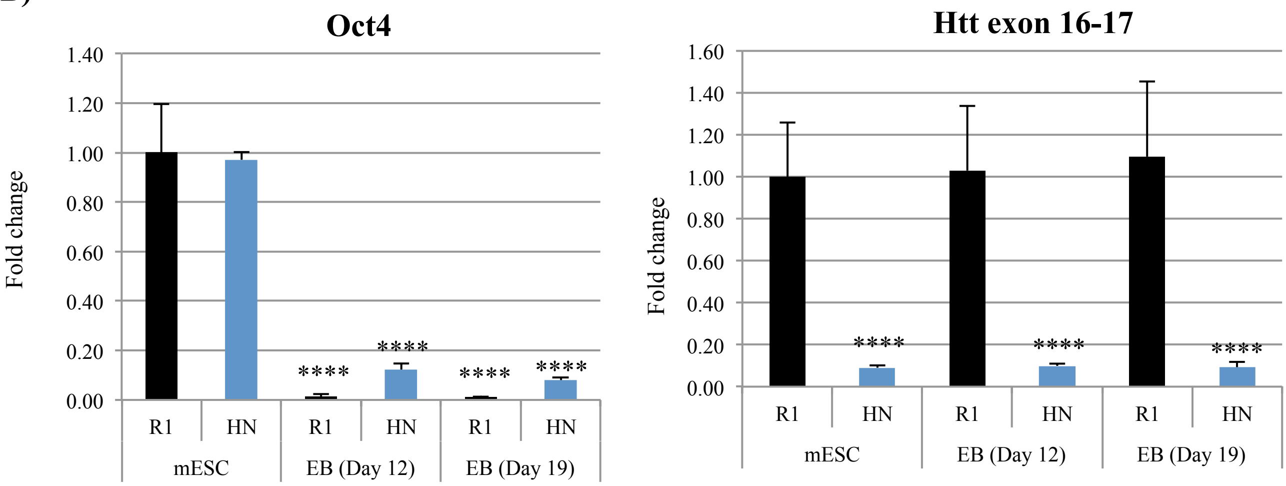

Supplementary Figure 3 (continued)

(C)

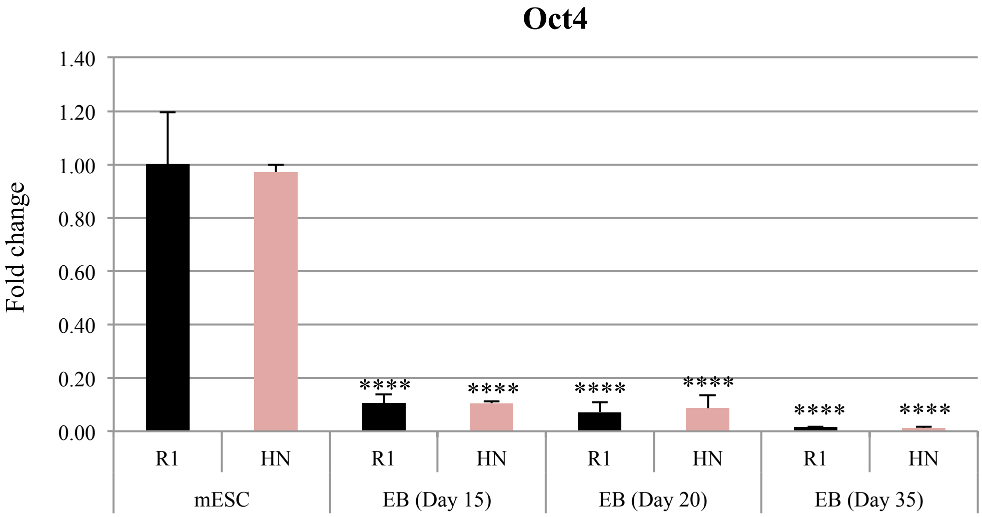

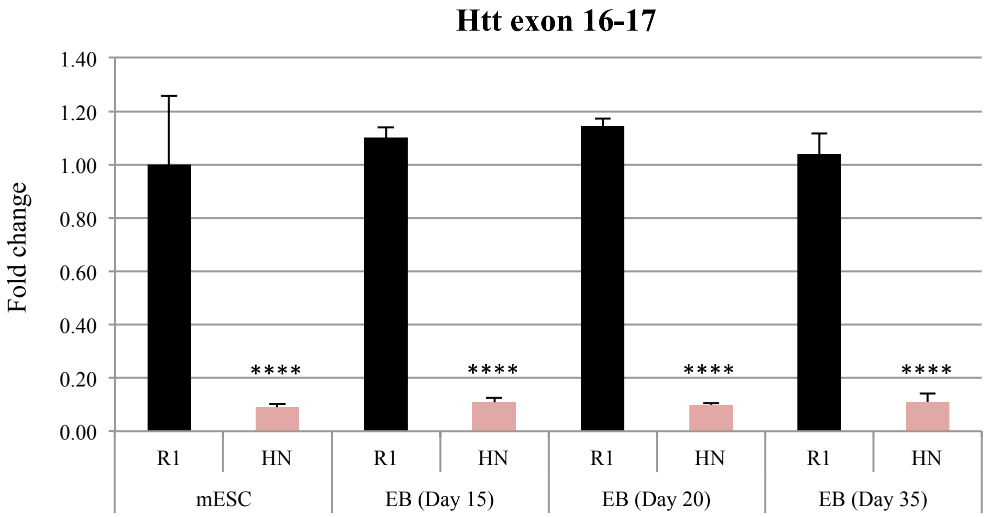

Supplement: Supplementary file 4 [file Image_3.PDF]
