# Supplementary material for: Huntingtin Is Required for Neural But Not Cardiac/Pancreatic Progenitor Differentiation of Mouse Embryonic Stem Cells In vitro
Source: Front Cell Neurosci. 2017 Feb 21;11:33. doi: 10.3389/fncel.2017.00033 (PMC5318384; doi:10.3389/fncel.2017.00033)

Supplementary Figure 4. Expression of Desmin in cardiac EBs. Immunocytochemistry shows both R1 and HN Day 12 cardiac EBs positive for Desmin staining. Scale bar: 25 µm

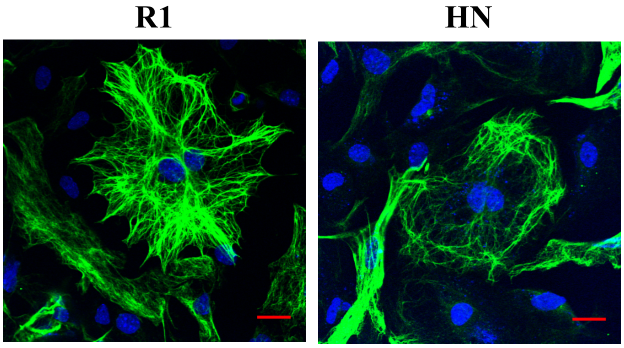

Supplement: Supplementary file 5 [file Image_4.PDF]
