# Supplementary material for: Huntingtin Is Required for Neural But Not Cardiac/Pancreatic Progenitor Differentiation of Mouse Embryonic Stem Cells In vitro
Source: Front Cell Neurosci. 2017 Feb 21;11:33. doi: 10.3389/fncel.2017.00033 (PMC5318384; doi:10.3389/fncel.2017.00033)

Supplementary Figure 5. Neural progenitor differentiation by the hanging-drop method starting with indicated number of cells. Scale bar: 200 μm

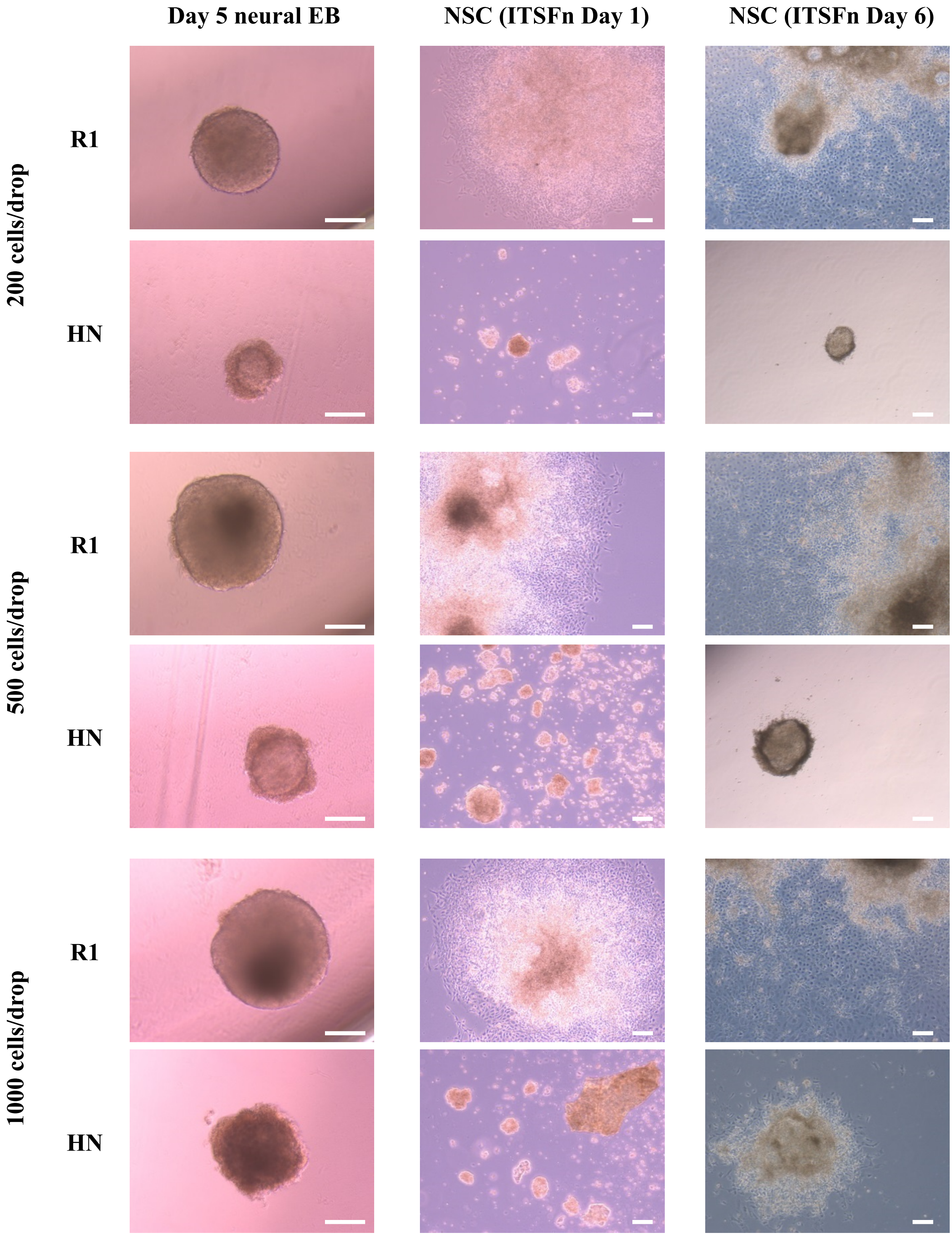

Supplement: Supplementary file 6 [file Image_5.PDF]
