# Supplementary material for: Huntingtin Is Required for Neural But Not Cardiac/Pancreatic Progenitor Differentiation of Mouse Embryonic Stem Cells In vitro
Source: Front Cell Neurosci. 2017 Feb 21;11:33. doi: 10.3389/fncel.2017.00033 (PMC5318384; doi:10.3389/fncel.2017.00033)

Supplementary Figure 6. Chromatin Immunoprecipitation analysis of Olig2 and Olig1 promoters. ChIP assays were carried out using chromatin extracted from R1 or HN mESCs and immuno-precipitated with rabbit α-Ezh2, mouse α-Htt2166, or mouse α-Htt (AbD Serotec, #MCA2050). Semi-quantitative PCR for Olig2 and Olig1 promoter region was performed. Occupancy of each protein was calculated as % input after measuring band intensity using the LI-COR Odyssey Fc Imaging System. ChIP antibodies used in the gel image are: Lane 1) rabbit IgG, 2) H3K27me3, 3) H3K4me3, 4) Htt2166, 5) Ezh2, 6) Pol II, 7) mouse IgG, 8) Serotec α-Htt, Lane 9) Input (10%).

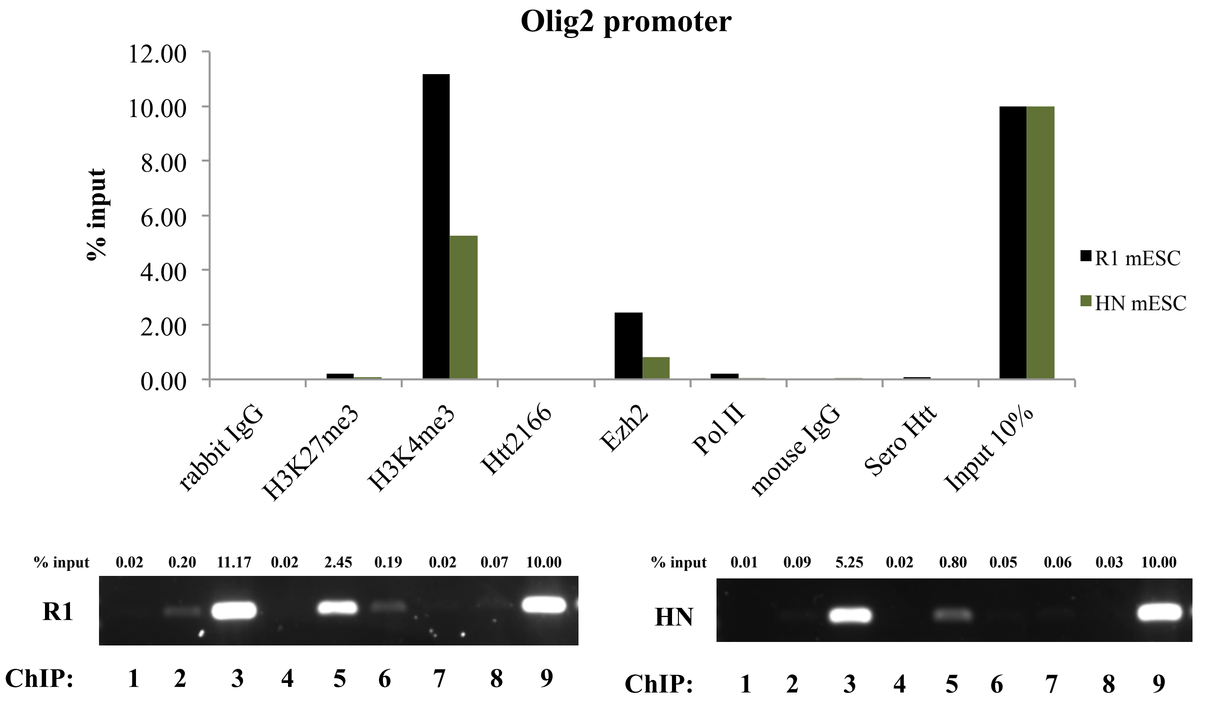

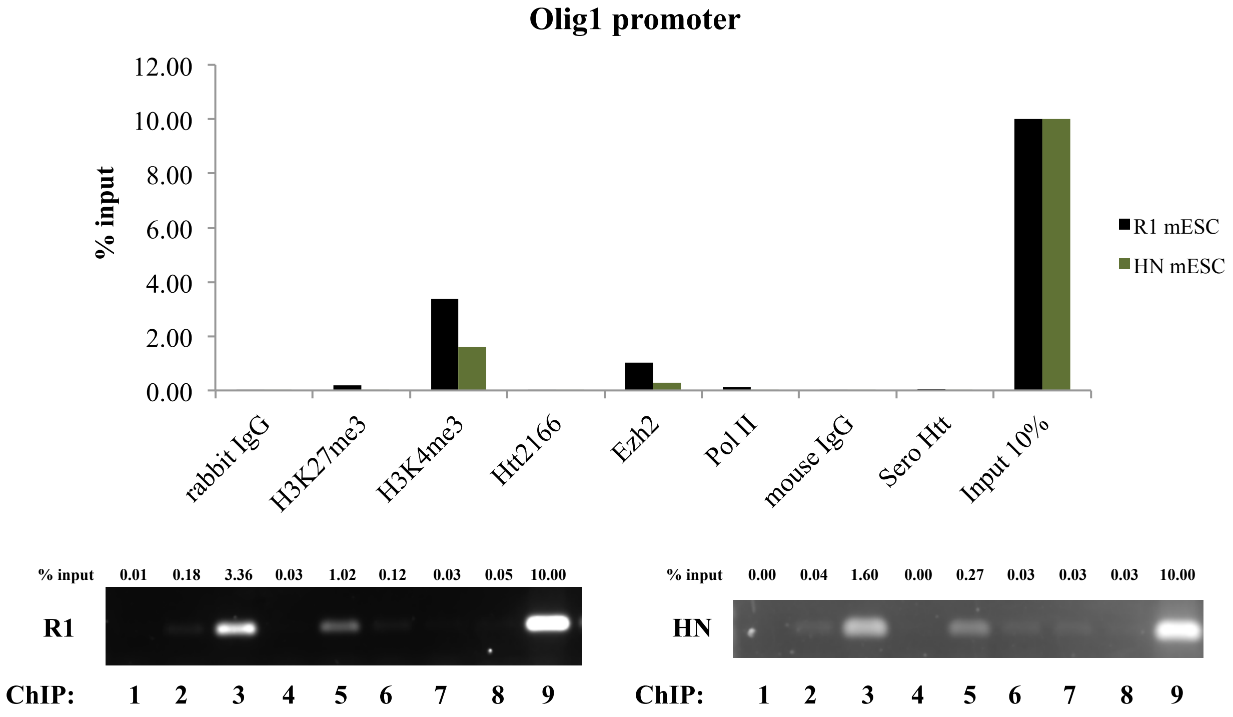

Supplement: Supplementary file 7 [file Image_6.PDF]
